# Chemical hydrodynamics of nuclear spin states

**DOI:** 10.1126/sciadv.ady9103

**Published:** 2025-10-22

**Authors:** Anupama Acharya, Madhukar Said, Sylwia J. Barker, Marcel Utz, Bruno Linclau, Ilya Kuprov

**Affiliations:** ^1^ School of Chemistry and Chemical Engineering, University of Southampton, University Road, Southampton SO17 1BJ, UK.; ^2^ Department of Organic and Macromolecular Chemistry, Ghent University, Krijgslaan 281-S4, 9000 Ghent, Belgium.; ^3^ Institute of Microstructure Technology, Karlsruhe Institute of Technology, Hermann-von-Helmholtz-Platz 1, Eggenstein-Leopoldshafen 76344, Germany.; ^4^ Department of Chemical and Biological Physics, Weizmann Institute of Science, 234 Herzl Street, Rehovot 76100, Israel.

## Abstract

Quantum mechanical equations of motion are strictly linear in density operators, but equations describing chemical kinetics and hydrodynamics may be nonlinear in concentrations. This incompatibility is fundamental, but special cases can be handled—for example, in magnetic resonance where nuclear spin interactions may be too weak influence concentration dynamics. For isolated spins and first-order reactions, this is a well-researched topic, but time evolution of complex nuclear spin systems in the presence of second-order kinetics, diffusion, and flow has so far remained intractable. In this communication, we report a numerically stable formalism for time-domain description of nuclear spin dynamics and relaxation in the simultaneous presence of diffusion, flow, and second-order chemical reactions. As an illustration, we use Diels-Alder cycloaddition of acrylonitrile to cyclopentadiene in the presence of diffusion and flow in a microfluidic NMR probe (a finite element model with thousands of Voronoi cells) with a spatially localized stripline radio frequency coil.

## Teaser

Microfluidic NMR is efficiently simulated in the simultaneous presence of nonlinear kinetics, diffusion, and hydrodynamics.

## INTRODUCTION

Fundamental equations of motion in quantum mechanics of isolated systems (*1*) and ensembles (*2*) are required, by causality and time translation invariance (*3*), to be linear with respect to state descriptors, such as wave functions and density matrices. However, the law of mass action in chemical kinetics (*4*) and Navier-Stokes equations in hydrodynamics (*5*, *6*) are not fundamental; they are statistical approximations and, therefore, at liberty to be nonlinear with respect to concentrations.

This incompatibility creates insidious difficulties in theoretical descriptions of systems where quantum processes coexist with chemical kinetics and spatial transport, notably in spin chemistry (*7*), magnetic resonance imaging (MRI) of complex metabolic (*8*) and hydrodynamic (*9*) processes, and—our predicament here—nuclear magnetic resonance (NMR) in microfluidic chips (*10*–*12*). The problem involves a collision of approximations at the interface of classical and quantum physics broadly similar to the measurement paradox (*13*). Although the concentration and the wave function amplitude square are both probability densities, one has a specific measurement outcome, but the other does not.

The general case has no solution; here we adopt a simplification from condensed-phase NMR and assume that nuclear spin processes are influenced by spatial dynamics and chemistry, but that there is no back action because nuclear spin interaction energies are very small. Processes like chemically induced dynamic nuclear polarisation (*14*) will unfortunately have to wait—there are raging debates about their magnetokinetics; here we focus on reactions that do not involve unpaired electrons. Another generally valid assumption in NMR of diamagnetic systems is that the electronic structure remains in the ground state, only manifesting through effective parameters of the nuclear spin Hamiltonian (*15*).

First-order kinetics (both exact and approximate) and nonreacting spatial transport of single spins in magnetic resonance are comprehensively researched and reviewed (*16*–*22*). Simulation of second-order kinetics has been looked at, but the best current formalism is not numerically friendly: Concentrations occur in denominators of the equations proposed by Kühne *et al.* (*21*), meaning that the common case of near-zero concentration yields a singularity that makes those equations numerically unstable. Simultaneous diffusion, flow, second-order kinetics, and fully quantum mechanical description of coherent and dissipative spin dynamics in large molecules have not been attempted due to the overwhelming numerical complexity of the task.

At the same time, such simulations are increasingly pertinent: Many systems studied by NMR and MRI involve chemical reactions. Lactate metabolism is one example: Increased lactate levels in mammalian cells (*23*) can be an indication of cancer and other disease (*24*, *25*). Increased pyruvate to lactate conversion is a symptom of inflammatory disease in the liver (*26*) and a sign of injury to the kidneys (*27*, *28*), as well as of diabetes (*29*). Another example is the tricarboxylic acid cycle: Anomalous intermediate concentrations can indicate cardiac and neurological disease (*30*–*32*). On the transport side, the simulation of spatially distributed, diffusing, and flowing systems is important in diffusion weighted imaging (*33*–*35*), diffusion tensor imaging (*36*), and vascular imaging by phase-contrast techniques (*37*–*39*). NMR is well integrated with these methods and used for quantification of metabolites (*40*–*42*). However, resolution and strong *J*-coupling problems in proton spectra have caused an exodus toward ^13^C and ^19^F NMR spectroscopy in metabolomics (*43*). Low natural abundance of ^13^C also produced a growing emphasis (*43*–*45*) on hyperpolarization techniques such as parahydrogenation (*46*, *47*) and dynamic nuclear polarisation (*48*, *49*). Pyruvate is a common target (*8*, *50*–*52*) as a key intermediate and a branching point for further metabolism (*32*). Much of the above has nonlinear kinetics.

No existing simulation software can handle this level of chemical, spatial, and spin dynamics complexity. Major existing packages, such as SIMPSON (*53*), GAMMA (*54*), and SpinEvolution (*55*), treat spin quantum mechanically but only cover solid orientation distributions. On the MRI side, packages such as SIMRI (*56*), coreMRI (*57*), JEMRIS (*58*), and MRISMUL (*59*) can model sophisticated spatial dynamics but use Bloch-Torrey equations (*60*) for spin. Very few MRI packages (*61*) implement Bloch-McConnell (*62*) solvers; most implementations are stand-alone simulation frameworks mainly focusing on chemical exchange saturation transfer MRI (*63*–*65*).

In this communication, we report a theoretical formalism and a Spinach (*66*) implementation for the full nonlinear kinetics + magnetohydrodynamics case. The problem is a quantum mechanical generalization of the Fokker-Planck formalism (*67*, *68*), in which concentration is replaced by concentration-weighted density matrix (*69*, *70*) and the evolution generator is both time and state dependent. Its efficient numerical implementation is difficult: Dimensions of spatial dynamics generator matrices on finite grids can be large; when combined with the spin Hamiltonian of a typical metabolite, the composite evolution generators cannot even be stored, let alone manipulated. This problem was recently solved by Allami *et al.* (*71*); we build on their methods by storing the combined evolution generators in a polyadic format with buffered Kronecker products. For logistical reasons also discussed in (*71*), the state vector remains uncompressed.

**Fig. 1.**
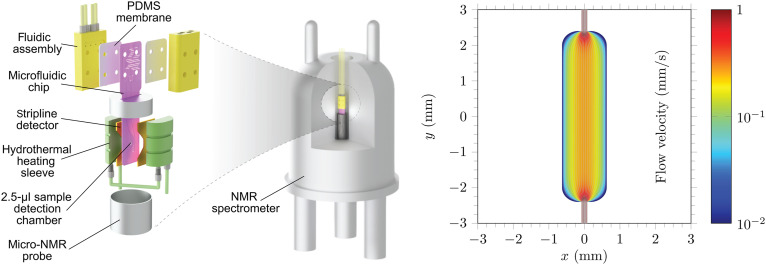
Microfluidic NMR experiment schematic. Microfluidic NMR is an example of a setting with simultaneous diffusion, hydrodynamics, chemical kinetics, and spatially distributed dissipative quantum dynamics in a multispin system. (**Left**) A schematic diagram of a microfluidic NMR probe (*72*) with a stripline radio-frequency coil (*12*). (**Right**) A finite volume simulation, using COMSOL (*74*), of the velocity field of the stationary fluid flow through the reaction chamber of the chip. PDMS, polydimethylsiloxane.

We apply the resulting software to microfluidic chip NMR experiments (Fig. 1) where we model simultaneous diffusion, flow, and spin dynamics during a second-order cycloaddition reaction. The chemistry and the engineering are described in (*72*, *73*): Reactants are delivered at the upper end of the chip, and the products are detected via their NMR signals at the lower end of the sample chamber. The spatial discretization mesh and the velocity field are imported from COMSOL (*74*). At the nuclear spin dynamics level, chemical kinetics is described using state-dependent superoperators (*70*) acting on concentration-weighted density matrices in each Voronoi cell of the mesh. Quantum mechanical treatment of coupled multispin systems, essential in such processes, is, therefore, maintained.

## EQUATIONS OF MOTION

In this section, we build a numerically friendly equation of motion for the concentration-weighted density matrix η=cρ under the assumption that kinetics, diffusion, and flow do not depend on the nuclear spin state. Chemical concentration c is a type of probability density and eigenvalues of the density matrix ρ are probabilities; eigenvalues of η are, therefore, also probability densities. The density matrix must be thermodynamically correct [i.e., the zero-trace tomfoolery (*75*, *76*) is not permitted] to reflect the fact that only a fraction of each substance is spin polarized; then, $c_{n}=\text{Tr}\left(\eta_{n}\right)$ , where the index n runs over chemical substances. The individual processes (kinetics, diffusion and flow, and spin dynamics) are described by Lie semigroup actions on the corresponding state spaces; we then merge their generator algebras (*70*, *77*) to bring them together.

### Chemical kinetics

Within the assumptions made by the law of mass action (*4*), a network of elementary chemical reactions involving N substances obeys the following equations

$$\frac{\partial c_{n}}{\partial t}=f_{n}\left(c_{1},\ldots ,c_{N}\right)$$ (1)

where $c_{n}$ is the concentration of *n*th substance, $f_{n}$ are low-order polynomials with location-dependent coefficients (for example, due to variations in temperature and ionic strength), and the partial derivative is a reminder that these equations govern local kinetics at each point of a three-dimensional (3D) sample. We assume that nuclear spin state has no effect on this dynamics.

### Diffusion and flow

We assume that Fick’s first Law (*78*) is also unaffected by the nuclear spin state

$$j_{n}=vc_{n}-D_{n}c_{n}\frac{\nabla \mu_{n}}{\mathrm{RT}}$$ (2)

where $j_{n}$ is the net flux of the *n*th substance, $D_{n}$ is its diffusion coefficient, v is the velocity of the fluid flow (may be time dependent), ∇ is the gradient operator with respect to the location within the sample, and $\mu_{n}$ is the chemical potential of the substance. For an ideal solution with uniform temperature and pressure, $\nabla \mu_{n}=\mathrm{RT}\nabla \text{ln}c_{n}=\left(\mathrm{RT}/c_{n}\right)\nabla c_{n}$ , and the equation simplifies into Fick’s original form that involves the concentration gradient

$$j_{n}=vc_{n}-D_{n}\nabla c_{n}$$ (3)

The continuity equation (*79*) then relates flux divergence to concentration change, and the kinetic terms are inherited from Eq. 1

$$\frac{\partial c_{n}}{\partial t}=-\nabla \cdot j_{n}+f_{n}\left(c_{1},\ldots ,c_{N}\right)$$ (4)

A logistical requirement at this stage is for the equation of motion to be expressed via concentrations rather than thermodynamic activities. This is because only concentrations are probability densities in the sense that is required for merging them with spin density operators later. In uniform solutions, activity coefficients may be absorbed into effective location- and concentration-dependent diffusion coefficients and reaction rates. We list the necessary assumptions in section S2 of the Supplementary Materials.

Up to this point, everything is standard (*80*); Eq. 4 may be solved independently. We therefore assume below that concentrations had been precomputed as functions of time and location; the particular numerical implementations used by Spinach are described in the “Implementation Details” section.

### Chemical transport of nuclear spin state

Concentrations can be zero or near-zero. For this reason, the formalism published by Kühne *et al.* (*21*) is unstable in finite precision arithmetic: Their equations 10 and 11 have concentrations in denominators. This was not a concern in 1979 when analytical solutions were the preferred way forward, but, here, we must build a formalism in which the computer never divides by a concentration and, preferably, never divides at all because the inverse might not exist under semigroup dynamics.

**Fig. 2.**
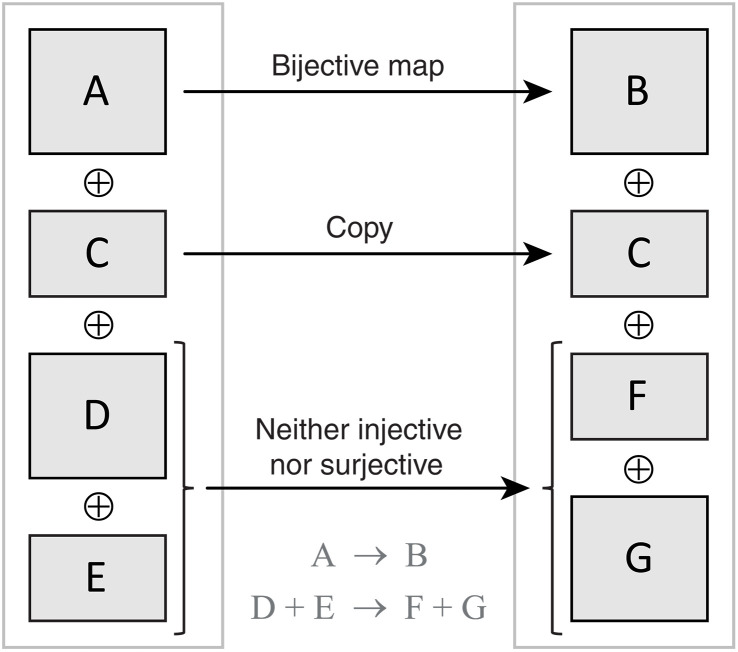
Maps between nuclear spin density matrix spaces. Three types of chemical processes are shown in a system containing substances {A, C, D, E} that are chemically converted into substances {B, C, F, G} as shown in the figure. Isomerization (A → B) changes the Hamiltonian but does not change the density matrix space; this is the well-researched case of a first-order chemical reaction going back to McConnell (*62*). Observer substances (for example, solvent) simply continue evolving under the same Hamiltonian (C → C). This paper resolves the implementation difficulties associated with bimolecular reactions such as (D + E → F + G) where the nuclear spin density matrix space on either side of the reaction arrow need not be the same: Some product states may be lost, some moved over, and some rearranged.

We must also account for the fact that spin state spaces may be different on either side of the reaction arrow; this is illustrated in Fig. 2. For example, the association process

A+B⇄C (5)

maps a direct sum of state spaces of reactants A and B (in a solution, reactant molecules would normally be uncorrelated in their nuclear spin state) into a direct product of those spaces that describes the substance C. The map induced by the forward reaction is clearly one-to-one, but the reverse reaction is a dissociation process: Some nuclear spin correlations in substance C would be broken up and lost because those two particular shards A and B are unlikely to meet again in a typical solution. Thorny complications that can arise here are debated in the spin chemistry literature (*81*–*83*). Those are only significant for electron spin dynamics; here, we deal with nuclei.

We assume that a nucleus, when moving from a reactant to a product, takes its spin state and ensemble correlations with it. In complete Liouville spaces that have a direct product structure (*84*), this assumption leads immediately to the kinetics superoperator (*17*). However, when an incomplete Liouville space is used to treat a large spin system (*85*) the procedure is more nuanced: We must consider individually the structure and the population of each state in the incomplete basis and account for the origin and the destination of each nucleus. For each chemical reaction, the kinetic part of the equation of motion is, therefore, built as described in (*70*). The algorithm is as follows:

1) Index the Liouville space basis $\left\{\;\beta_{p}^{\left(n\right)}\;\right\}$ of nuclear spin states for every substance involved in the reaction. Here, n runs over substances and p over basis states. Each basis state $\beta_{p}^{\left(n\right)}$ is a direct product of single-nucleus spin operators of the form

$$\begin{array}{c} T_{l_{1},m_{1}}⊗T_{l_{2},m_{2}}⊗T_{l_{3},m_{3}}⊗\cdots \\ ⇕\\ \left(\begin{array}{cccc} l_{1} & l_{2} & l_{3} & \cdots \\ m_{1} & m_{2} & m_{3} & \cdots \end{array}\right) \end{array}$$ (6)

where l and m are state indices of the single-spin irreducible spherical tensor basis set (*15*). Only the indices need to be stored by a computer because they define the operator unambiguously. In large spin systems, this basis may be incomplete because some states are dynamically unreachable (*85*).

2) Using the indexed representation in Eq. 6, build a matching table of nuclear spin basis states on either side of the reaction arrow, indicating which state on the left is mapped into which state on the right. The following is possible: (i) an identical state involving the same spins exists in the destination basis; its population is then to be drained from the source space and replenished in the destination space; (ii) an identical state involving the same spins does not exist in the destination basis, for example, because it becomes intermolecular; its population is then to be drained from the source space but not forwarded to any destination.

The result is a set of superoperators that we call drain generators $D_{\mathrm{rs}}$ (sparse matrices with −1 on the diagonal for each state that is drained by reaction r from substance s ) and fill generators $F_{\mathrm{rs}}$ (sparse matrices with +1 between the source and the destination whenever the destination exists in the basis set). These matrices are evolution generators in the Lie semigroup sense: To produce propagators acting on the system state vector, they need to be multiplied by the corresponding rates, added up, and exponentiated with the chosen time step.

3) At each point of the sample, the local concentration-weighted density matrix $\eta_{n}$ of each substance n then evolves according to the following equation

$$\begin{array}{c} \begin{array}{c} \frac{\partial }{\partial t}\left(\begin{array}{c} \eta_{1}\\ \eta_{2}\\ \vdots \end{array}\right)=-i\left(\begin{array}{c} H_{1}\left(r,t\right)\eta_{1}\\ H_{2}\left(r,t\right)\eta_{2}\\ \vdots \end{array}\right)+\left(\begin{array}{c} R_{1}\left(r,t\right)\eta_{1}\\ R_{2}\left(r,t\right)\eta_{2}\\ \vdots \end{array}\right)+\\ +\sum_{r}k_{r}\sum_{s\in r}\left(\prod_{m\neq s}c_{m}\right)\left(D_{\mathrm{rs}}+F_{\mathrm{rs}}\right)\left(\begin{array}{c} \eta_{1}\\ \eta_{2}\\ \vdots \end{array}\right) \end{array} \end{array}$$ (7)

where Hamiltonian commutation superoperators $H_{n}$ may be time- and location-dependent. Relaxation superoperators $R_{n}$ may be location-dependent [for example, due to variations in local viscosity (*86*, *87*), magnetic field, or other parameters] but are not usually intrinsically time-dependent. In the kinetics superoperator, the outer sum is over the chemical reactions, and $k_{r}$ are their rate constants from the mass action law. The s index of the inner sum enumerates reactants; each of them forward its spin states to the products as prescribed by the drain generator $D_{\mathrm{rs}}$ and the fill generator $F_{\mathrm{rs}}$ . The rate of that process is proportional to the concentration product, but reactant’s own concentration is already present in the concentration-weighted density matrix—therefore, only the remaining concentrations are multiplied up.

At each spatial location, Eq. 7 describes nuclear spin dynamics driven by coherent evolution, relaxation, and chemical redistribution of nuclear spin state populations. It holds simultaneously with Eq. 4 that governs spatial dynamics and chemical kinetics. They are connected by the concentration dependence of the chemical transport rates and the fact that the physical quantity seen by the NMR instrument is the concentration-weighted density matrix.

### Spatial transport of nuclear spin state

When substances with nonequilibrium nuclear spin states are introduced into the system, they are moved around by diffusion and flow. We also assume that spatial transport does not depend on the nuclear spin state; this is exceedingly well studied in the Bloch equation limit (*88*–*90*). For the density matrix, the equation enforcing the conservation of probability reads

$$\frac{\partial \eta_{n}}{\partial t}=-\nabla \cdot \left(v⊗\eta_{n}-D_{n}\nabla \eta_{n}\right)$$ (8)

where two terms in the brackets are fluxes of η due to flow and diffusion. It reflects the fact that concentration gradients are not required for the density matrix transport to occur: Spin states still move around even if concentrations are uniform. Equation 8 does not require us to keep track of concentration and spin state independently; only their product is needed, a significant logistical improvement over the state of the art (*21*).

### Combined equations of motion

With individual components now in place, we must solve two systems of partial differential equations. First, the nuclear spin–independent diffusion, flow, and chemical kinetics

$$\begin{array}{c} \frac{\partial c_{n}}{\partial t}=-\nabla \cdot \left[v\left(r,t\right)c_{n}-D_{n}\nabla c_{n}\right]+f_{n}\left(c_{1},\ldots ,c_{N}\right) \end{array}$$ (9)

where initial and boundary conditions are specified by the user. This stage is well researched (*80*); after solving Eq. 9, we get time and location dependence of all concentrations $c_{n}\left(r,t\right)$.

The second system of equations is the balance of probability for the concentration-weighted density matrix of each substance, including all evolution generators discussed above

$$\begin{array}{c} \begin{array}{c} \frac{\partial }{\partial t}\left(\begin{array}{c} \eta_{1}\\ \eta_{2}\\ \vdots \end{array}\right)=-\nabla \cdot \left(\begin{array}{c} v⊗\eta_{1}-D_{1}\nabla \eta_{1}\\ v⊗\eta_{2}-D_{2}\nabla \eta_{2}\\ \vdots \end{array}\right)-i\left(\begin{array}{c} H_{1}\left(r,t\right)\eta_{1}\\ H_{2}\left(r,t\right)\eta_{2}\\ \vdots \end{array}\right)\\ +\left(\begin{array}{c} R_{1}\left(r,t\right)\eta_{1}\\ R_{2}\left(r,t\right)\eta_{2}\\ \vdots \end{array}\right)+\sum_{r}k_{r}\sum_{s\in r}\left(\prod_{m\neq s}c_{m}\right)\left(D_{\mathrm{rs}}+F_{\mathrm{rs}}\right)\left(\begin{array}{c} \eta_{1}\\ \eta_{2}\\ \vdots \end{array}\right) \end{array} \end{array}$$ (10)

where elements of column vectors refer to different substances. Spin evolution generators can act on η instead of ρ because they commute with the scalar concentration multiplier. The list of assumptions pertaining to this equation is given in section S2 of the Supplementary Materials.

Relaxation superoperators $R_{n}$ in Eq. 10 are rarely explicitly time dependent but commonly location dependent; that is accounted for by assigning a different relaxation superoperator to each cell of the mesh. A more subtle matter is that the thermal equilibrium state is concentration dependent

$$\eta_{n}^{\left(\text{eq}\right)}=c_{n}\text{exp}\left(-H_{n}/\mathrm{kT}\right)/\text{Tr}\left[\text{exp}\left(-H_{n}/\mathrm{kT}\right)\right]$$ (11)

In the conventional formulation of the Liouville space, as the adjoint representation of the Hilbert space (*84*), this would require the relaxation superoperator to be updated every time concentration changes, an inconvenient and numerically expensive process. However, the indexed product state basis sets used by Spinach (*85*) have the trace of the density matrix as the first element of the state vector. When concentration weighting is then performed, the first element of the state vector ends up being concentration, meaning that concentration-dependent thermalization within the inhomogeneous master equation formalism (*91*) takes care of itself

$$\begin{array}{c} \begin{array}{c} \begin{array}{c} \frac{\partial \rho }{\partial t}=\ldots +R\left(\rho -\rho_{\text{eq}}\right)\;\Rightarrow \;\frac{d}{\mathrm{dt}}\left(\begin{array}{c} 1\\ \rho \end{array}\right)=\ldots +\left[\begin{array}{cc} 0 & 0\\ -R\rho_{\text{eq}} & R \end{array}\right]\left(\begin{array}{c} 1\\ \rho \end{array}\right)\\ \frac{\partial c\rho }{\partial t}=\ldots +R\left(c\rho -c\rho_{\text{eq}}\right)\;\Rightarrow \;\frac{d}{\mathrm{dt}}\left(\begin{array}{c} c\\ c\rho \end{array}\right)=\ldots +\left[\begin{array}{cc} 0 & 0\\ -R\rho_{\text{eq}} & R \end{array}\right]\left(\begin{array}{c} c\\ c\rho \end{array}\right) \end{array} \end{array} \end{array}$$ (12)

Note that the operator in square brackets is the same in both lines; this automatic concentration scaling behavior is a welcome feature—it means that each relaxation superoperator needs only to be thermalized once to drive the system to

$$\rho_{n}^{\left(\text{eq}\right)}=\text{exp}\left(-H/\mathrm{kT}\right)/\text{Tr}\left[\text{exp}\left(-H/\mathrm{kT}\right)\right]$$ (13)

This approach requires a segmented basis set in which every chemical substance has a separate unit state; basis generation utility in Spinach kernel takes care of that automatically.

As required, and unlike the formalism presented in (*21*), there are no denominators in Eq. 10; it is, therefore, stable in finite precision arithmetic when concentrations are close to zero. It is solved using standard numerical methods discussed in the “Implementation details” section below; we recommend Lie quadratures that respect group-theoretical constraints (*92*–*95*). They were also recently implemented in Spinach (*96*).

## IMPLEMENTATION DETAILS

Coding up a solver and visualization tools for Eqs. 9 and 10 is a harder exercise than the straightforward mathematical derivation that we just gave it. Omitting software implementation details is something theory papers are too often guilty of; we break with that tradition here.

### Mesh and flow velocity data handling

**Fig. 3.**
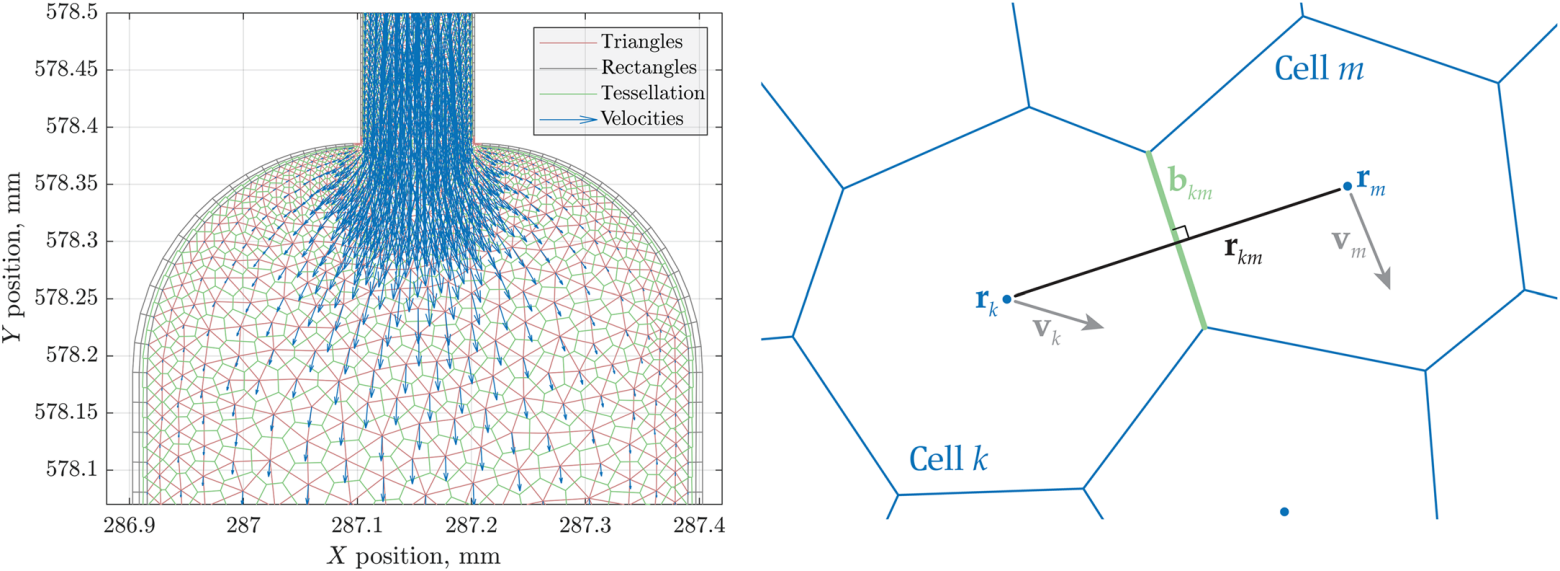
A numerical model of the sample chamber of the microfluidic chip. An adaptive grid in the left panel (red lines) was produced by COMSOL (*74*). Blue arrows indicate stationary flow velocities and green lines are Voronoi tessellation produced by Matlab (*97*). Solutions of the flow and the chemistry problems are handled by COMSOL; stationary velocity fields are then imported into Spinach 2.11 (*66*) that handles the nuclear spin dynamics as described in the main text. The right panel shows finite volume hydrodynamics solver setup schematic in two dimensions. Blue lines are edges of Voronoi tessellation cells, blue dots are cell centers, and gray arrows are velocity vectors. The local rate of change in substance concentration in each cell is computed as a balance of advective and diffusive fluxes through cell boundaries (see the “Discretization of the equation of motion” section).

Matlab does have a meshing tool for arbitrary domains (*97*), but, in our context, it was more convenient to import the mesh from COMSOL (*74*) because that is where the stationary flow velocity field had been computed by the experimental team. Plain text files containing mesh information were parsed and mesh elements classified into edges, triangles, and rectangles; the latter are used by COMSOL to indicate impenetrable walls (Fig. 3, left).

Spinach stores mesh information as a data structure (fig. S3 in the Supplementary Materials) with the fields containing vertex coordinates and three index arrays: pairs of integers indicating which vertices make an edge, triads of integers for triangles, and tetrads of integers for rectangles. Voronoi tessellation information, computed by Matlab, is added as further arrays with coordinates of Voronoi vertices, integer indices for which vertices belong to which Voronoi cell, and weights indicating the volume of each cell.

On the visualization side, Matlab figures are objects—when a new element is added, a new sub-object is created. It is, therefore, impractical to draw complex meshes line by line—the list of sub-objects becomes too large for interactive plotting. For this reason, further arrays are precomputed, containing Cartesian coordinates of the endpoints of each line separated by NaN values. When Matlab encounters NaN values in coordinate arrays, it creates line breaks; this allows the entire mesh to be plotted as one object which accelerates interactive graphics. All of the above arrays are stored in the .mesh subfield of the Spinach spin_system data structure (fig. S3 in the Supplementary Materials).

### Discretization of the equation of motion

A gridded domain, such as that in the left panel of Fig. 3, has a set of locations corresponding to Voronoi cells numbered by the index k with centers at $r_{k}$ . Each location has a set of substance concentrations $c_{\mathrm{kn}}$ (first index refers to location and second to the substance) and a set of flux vectors $j_{\mathrm{kn}}$ . Each substance at each location has a nuclear spin density matrix $\rho_{\mathrm{kn}}$ with a unit trace. The objective is to calculate the dynamics of concentration-weighted density matrices $\eta_{\mathrm{kn}}=c_{\mathrm{kn}}\rho_{\mathrm{kn}}$.

We import the (possibly, time dependent) flow velocity field from specialized software, in this case COMSOL (*74*). Without the reaction terms already discussed above, the continuous forms of Eqs. 2 and 4 for each substance in a stationary flow and diffusion regime are

∂c(r,t)/∂t=−∇⋅j(r,t) (14)

j(r,t)=v(r)c(r,t)−D∇c(r,t) (15)

where the flux vector j(r,t) has an advection component from the flow velocity field v(r) and a diffusion component proportional to the concentration gradient under the assumptions listed in section S2 of the Supplementary Materials.

The standard finite volume algorithm (a schematic for the 2D case is shown in the right panel of Fig. 3), is essentially a set of conservation laws that balance local concentration changes with boundary integrals of fluxes (*98*). For didactic purposes, we include here a derivation for the 2D case; the 3D case may be found in the hydrodynamics literature (*99*, *100*).

We start by integrating Eq. 14 over the area $A_{k}$ of the Voronoi cell k and note that the time derivative on the left-hand side commutes with area integration

$$\frac{\partial }{\partial t}\iint_{A_{k}}c\;\mathrm{dA}=-\iint_{A_{k}}\nabla \cdot j\;\mathrm{dA}$$ (16)

After we apply the divergence theorem (*101*) on the right-hand side, this becomes

$$\frac{\partial }{\partial t}\iint_{A_{k}}c\;\mathrm{dA}=-\oint_{B_{k}}j\cdot n\;\mathrm{dB}$$ (17)

where $B_{k}$ is the boundary of the Voronoi cell k and n is the outward normal vector. On the left-hand side, we now assume that the mesh is fine enough for the concentration $c_{k}$ to be constant within each cell k . On the right-hand side, we break the contour integral up into integrals along each boundary segment of the cell (the right panel of Fig. 3 illustrates the notation)

$$\begin{array}{c} A_{k}\frac{\partial c_{k}}{\partial t}=-\sum_{m\in N_{k}}\int_{B_{\mathrm{km}}}j\cdot n_{\mathrm{km}}\mathrm{dB},\;n_{\mathrm{km}}=\frac{r_{\mathrm{km}}}{|\;r_{\mathrm{km}}\;|} \end{array}$$ (18)

where $N_{k}$ is the set of neighboring cells to cell k , $B_{\mathrm{km}}$ is the shared boundary of cells k and m , and the expression for $n_{\mathrm{km}}$ follows from the definition of Voronoi tessellation (*102*, *103*). We now proceed to use Eq. 15 and approximate flux integrals through each boundary as

$$\begin{array}{c} \int_{B_{\mathrm{km}}}\left(vc\right)\cdot n_{\mathrm{km}}\mathrm{dB}\;\approx \;|\;b_{\mathrm{km}}\;|\frac{v_{k}c_{k}+v_{m}c_{m}}{2}\cdot \frac{r_{\mathrm{km}}}{|\;r_{\mathrm{km}}\;|} \end{array}$$ (19)

$$\int_{B_{\mathrm{km}}}\left(D\nabla c\right)\cdot n_{\mathrm{km}}\mathrm{dB}\approx D|\;b_{\mathrm{km}}\;|\frac{c_{m}-c_{k}}{|\;r_{\mathrm{km}}\;|}$$ (20)

where $|b_{\mathrm{km}}|$ is the length of the shared boundary of cells k and m , and the flux is the average flux at the boundary of the two neighboring cells. After cosmetic rearrangements

$$\begin{array}{c} \frac{\partial c_{k}}{\partial t}=\sum_{m\in N_{k}}\frac{D|\;b_{\mathrm{km}}\;|}{A_{k}}\frac{c_{m}-c_{k}}{|\;r_{\mathrm{km}}\;|}-\sum_{m\in N_{k}}\frac{|\;b_{\mathrm{km}}\;|}{A_{k}}\frac{v_{k}c_{k}+v_{m}c_{m}}{2}\cdot \frac{r_{\mathrm{km}}}{|\;r_{\mathrm{km}}\;|} \end{array}$$ (21)

In a matrix representation, for the spatial transport generator F acting on a column vector c of substance concentrations in each Voronoi cell

$$\begin{array}{c} \begin{array}{c} F_{\mathrm{km}}=\left\{\begin{array}{cc} \frac{1}{A_{k}}\frac{|\;b_{\mathrm{km}}\;|}{|\;r_{\mathrm{km}}\;|}\left(D-\frac{v_{m}\cdot r_{\mathrm{km}}}{2}\right) & m\in N_{k}\\ 0 & m\notin N_{k} \end{array}\right.\\ F_{\mathrm{kk}}=-\sum_{m\neq k}F_{\mathrm{mk}},\;\frac{dc}{\mathrm{dt}}=Fc \end{array} \end{array}$$ (22)

where $F_{\mathrm{kk}}$ are computed using the conservation of matter balance because it must be enforced to machine precision. Examples of concentration evolution under this equation are shown in the “Diffusion and flow” section; annotated Matlab code is released as a part of the open-source Spinach library (*66*).

When the flux generator F acts on the concentration-weighted density matrix η , it should be extended to a Kronecker product F⊗1 that acts with a unit matrix on the spin subspace because the dynamics that it generates is nuclear spin independent. With that in place, continuous degrees of freedom are now discretized, and Eq. 10 acquires a pure matrix-vector form

$$\begin{array}{c} \begin{array}{c} \frac{\text{∂}}{\text{∂}t}\left(\begin{array}{c} \eta_{1}\\ \eta_{2}\\ \vdots \end{array}\right)=\left(\begin{array}{c} \left(F⊗1\right)\eta_{1}\\ \left(F⊗1\right)\eta_{2}\\ \vdots \end{array}\right)-i\;\left(\begin{array}{c} H_{1}\left(r,t\right)\eta_{1}\\ H_{2}\left(r,t\right)\eta_{2}\\ \vdots \end{array}\right)\;+\\ +\;\left(\begin{array}{c} R_{1}\left(r,t\right)\eta_{1}\\ R_{2}\left(r,t\right)\eta_{2}\\ \vdots \end{array}\right)+\sum_{r}k_{r}\sum_{s\in r}\left(\prod_{m\neq s}c_{m}\left(r,t\right)\right)\left(D_{\mathrm{rs}}+F_{\mathrm{rs}}\right)\left(\begin{array}{c} \eta_{1}\\ \eta_{2}\\ \vdots \end{array}\right) \end{array} \end{array}$$ (23)

where diffusion, flow, spin evolution, and relaxation happen independently for each chemical substance. The corresponding blocks of the equation of motion are linked by chemical transport of nuclear spin states with concentration-dependent rates. We recommend Lie group solvers (*93*, *104*) for this equation with the standard health warnings about time discretization step being small enough for the corresponding product quadratures to converge.

### Chemical reaction specification

Spin system specification in Spinach is described in detail in the program documentation; simple tutorials are also available in our recent paper (*105*); here, we focus on the syntax associated with the nonlinear kinetics and on the way that chemical reaction generators $D_{\mathrm{rs}}$ and $F_{\mathrm{rs}}$ in Eq. 23 are built in the actual code. The first logistical hurdle is the need to reindex interaction arrays when multiple noninteracting spin systems are brought together into a single data structure.

To that end, we have implemented a merge_inp function in Spinach 2.11 that combines and reindexes multiple sys and inter input structures (*105*) automatically

[sys,inter]= merge_inp({sys_a,sys_b},{inter_a,inter_b})

and also extends the rotational correlation time variable to an array that holds one rotational correlation time (a scalar or a tensor when rotational diffusion is anisotropic) per chemical species. That may be necessary, for example, when viscosity is different on either side of the cellular membrane in a translocation process (*86*, *87*). In that case, the relaxation superoperator can vary between compartments and the membrane translocation process is best handled as a local chemical reaction rather than position-dependent diffusion coefficient.

**Fig. 4.**
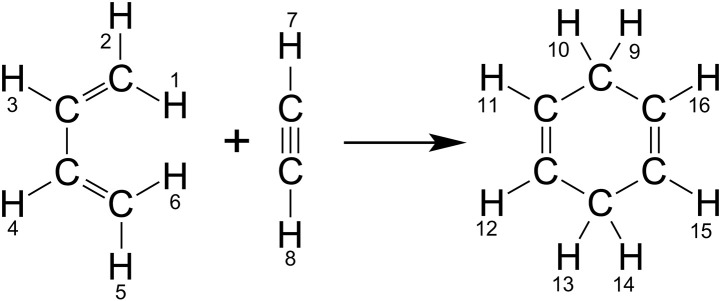
A simple Diels-Alder cycloaddition reaction. Sequential proton numbering is used for the matching of source and destination spin states on either side of the reaction arrow as described in the main text.

Chemical species indexing in Spinach is unchanged since the description given in (*105*). Consider a simple cycloaddition reaction involving ^12^C carbons so that only protons have spin (Fig. 4). For this reaction, the part specification is a cell array of integer sequences

inter.chem.parts = {1:6, 7:8, 9:16};

It indicates that there are three chemical species: The first one contains spins 1 to 6, the second one contains spins 7 and 8, and the third one contains spins from 9 to 16. These protons and their corresponding interactions are expected to have been declared earlier in the input stream as described in (*105*).

We now come to the input structure in Spinach 2.11, called reaction, that supports nonlinear kinetics. Its reactants subfield is an array of integers specifying which chemical species are on the left side of the reaction arrow; the products subfield does the same for the chemical species on the right side. For the cycloaddition reaction above

reaction.reactants = [1 2];

reaction.products = [3];

The information about which spin in the reactant set becomes which spin in the product set is provided as a matching table with pairs of integers. For the cycloaddition reaction above

reaction.matching = [1 9; 2 10; 3 11; 4 12; 5 13; 6 14; 7 16; 8 15];

Combined with the basis set specification built as described in (*85*), this allows Spinach to build reaction drain and fill generators as described in the “Chemical transport of nuclear spin state” section in a single user command

G = react_gen(spin_system,reaction);

When multiple reactions are present in the system, this function is called multiple times with different reaction specifications to obtain the corresponding matrices $D_{\mathrm{rs}}$ and $F_{\mathrm{rs}}$ that are returned, as elements of the cell array G, by react_gen.m function. As discussed in the “Chemical transport of nuclear spin state” section, they are drain and fill generators in the Lie algebraic sense: They do not depend on concentrations or time—they only indicate what goes where in the spin state space of the system as a result of each reaction. Concentration dependence turns up in their coefficients in Eq. 10.

A subtle point is that the cycloaddition reaction above yields not one but four products: one for acetylene binding from the top, one from the bottom, and two across. In the absence of selective isotope labeling, such topological matters are inconsequential to a chemist, but they may be significant to an NMR spectroscopist in the rare cases where the reactants have nonsymmetric nuclear spin states. In those cases, multiple reactions with different matching tables must be specified.

### Generator and state vector structure

For logistical reasons (to do with object layouts in Matlab), Spinach uses the following order of direct products in evolution generators that have spatial, chemical, and spin degrees of freedom (*71*)

$$G\left(t\right)=\sum_{\mathrm{nmk}}g_{\mathrm{nmk}}\left(t\right)M_{n}⊗K_{m}⊗S_{k}$$ (24)

where $g_{\mathrm{nmk}}\left(t\right)$ are interaction coefficients, $M_{n}$ are spatial dynamics generators, $K_{m}$ are chemical kinetics generators, and $S_{k}$ are spin dynamics generators. Accordingly, the initial state vector $\eta \left(t_{0}\right)$ is built from concentrations and spin states of each substance in the following way

$$\eta \left(t_{0}\right)=\left[\begin{array}{c} \left(\begin{array}{c} c_{1}^{\left(1\right)}\left(t_{0}\right)\\ \vdots \\ c_{N}^{\left(1\right)}\left(t_{0}\right) \end{array}\right)⊙\left(\begin{array}{c} \rho_{1}^{\left(1\right)}\left(t_{0}\right)\\ \vdots \\ \rho_{N}^{\left(1\right)}\left(t_{0}\right) \end{array}\right)\\ \vdots \\ \left(\begin{array}{c} c_{1}^{\left(K\right)}\left(t_{0}\right)\\ \vdots \\ c_{N}^{\left(K\right)}\left(t_{0}\right) \end{array}\right)⊙\left(\begin{array}{c} \rho_{1}^{\left(K\right)}\left(t_{0}\right)\\ \vdots \\ \rho_{N}^{\left(K\right)}\left(t_{0}\right) \end{array}\right) \end{array}\right]$$ (25)

where ⊙ denotes elementwise multiplication, $c_{n}^{\left(k\right)}$ is the concentration of the *n*th substance in the *k*th Voronoi cell of the mesh, and $\rho_{n}^{\left(k\right)}$ is the corresponding nuclear spin density matrix represented by a vector in a full or restricted Liouville space. There are no multiplicative ambiguities here because density matrices have unit traces.

When the state vector is evolved in time under Eq. 23, concentrations and density matrices can no longer be separated without loss of numerical stability, and, therefore, only products $\eta_{n}^{\left(k\right)}=c_{n}^{\left(k\right)}\rho_{n}^{\left(k\right)}$ are stored in the trajectory array T at each time discretization point $t_{m}$

$$T=\left[\begin{array}{cccc} \left(\begin{array}{c} \eta_{1}^{\left(1\right)}\left(t_{0}\right)\\ \vdots \\ \eta_{N}^{\left(1\right)}\left(t_{0}\right) \end{array}\right) & \left(\begin{array}{c} \eta_{1}^{\left(1\right)}\left(t_{1}\right)\\ \vdots \\ \eta_{N}^{\left(1\right)}\left(t_{1}\right) \end{array}\right) & \left(\begin{array}{c} \eta_{1}^{\left(1\right)}\left(t_{2}\right)\\ \vdots \\ \eta_{N}^{\left(1\right)}\left(t_{2}\right) \end{array}\right) & \cdots \\ \vdots & \vdots & \vdots & \cdots \\ \left(\begin{array}{c} \eta_{1}^{\left(K\right)}\left(t_{0}\right)\\ \vdots \\ \eta_{N}^{\left(K\right)}\left(t_{0}\right) \end{array}\right) & \left(\begin{array}{c} \eta_{1}^{\left(K\right)}\left(t_{1}\right)\\ \vdots \\ \eta_{N}^{\left(K\right)}\left(t_{1}\right) \end{array}\right) & \left(\begin{array}{c} \eta_{1}^{\left(K\right)}\left(t_{2}\right)\\ \vdots \\ \eta_{N}^{\left(K\right)}\left(t_{2}\right) \end{array}\right) & \cdots \end{array}\right]$$ (26)

This array may be stored explicitly or (for efficiency reasons) only the previous state vector may be kept at each point in the time-domain simulation.

The detection state δ is built in the same way as the initial condition in Eq. 25, but the role of concentration is played by the $B_{+}$ map of each radio frequency coil. It may be different in different parts of the sample because the coil field vector may be different

$$\delta =\left(\begin{array}{c} b_{+}^{\left(1\right)}\\ \vdots \\ b_{+}^{\left(K\right)} \end{array}\right)⊗\left(\begin{array}{c} L_{+}^{\left(1\right)}\\ \vdots \\ L_{+}^{\left(N\right)} \end{array}\right)=\left[\begin{array}{c} \left(\begin{array}{c} b_{+}^{\left(1\right)}L_{+}^{\left(1\right)}\\ \vdots \\ b_{+}^{\left(1\right)}L_{+}^{\left(N\right)} \end{array}\right)\\ \vdots \\ \left(\begin{array}{c} b_{+}^{\left(K\right)}L_{+}^{\left(1\right)}\\ \vdots \\ b_{+}^{\left(K\right)}L_{+}^{\left(N\right)} \end{array}\right) \end{array}\right]$$ (27)

where $b_{+}^{\left(k\right)}$ is the receptivity of the coil in the *k*th Voronoi cell of the mesh and $L_{+}^{\left(n\right)}=L_{X}^{\left(n\right)}+iL_{Y}^{\left(n\right)}$ is the Liouville space representation of the quadrature detection operator of the *n*th substance. Taking the inner product of δ with the trajectory array (but note the absence of complex conjugation in O=Tr[Oρ] ) yields a quantity proportional to the voltage induced in the detection coil

$$\begin{array}{c} \begin{array}{c} \langle \delta |T=\sum_{\mathrm{kn}}b_{+}^{\left(k\right)}\left[\text{Tr}\left(L_{+}^{\left(n\right)}\eta_{n}^{\left(k\right)}\left(t_{0}\right)\right)\;\text{Tr}\left(L_{+}^{\left(n\right)}\eta_{n}^{\left(k\right)}\left(t_{1}\right)\right)\;\text{Tr}\left(L_{+}^{\left(n\right)}\eta_{n}^{\left(k\right)}\left(t_{2}\right)\right)\;\cdots \right] \end{array} \end{array}$$ (28)

in which the observable is weighted with both the concentration (through the use of concentration-weighted density matrix) and the coil receptivity (through the use of the coil map).

Block structure of evolution generators matches the state vector layout described above, taking into account location and concentration dependence of the individual terms. Hamiltonian and relaxation superoperators may be different for each substance n in each Voronoi cell k ; they are, therefore, built from “phantoms” pertaining to individual interactions and relaxation mechanisms

$$\begin{array}{c} H_{n}^{\left(k\right)}=\sum_{j}\varphi_{\mathrm{nkj}}^{\left(H\right)}B_{\mathrm{nj}}^{\left(H\right)},\;R_{n}^{\left(k\right)}=\sum_{j}\varphi_{\mathrm{nkj}}^{\left(R\right)}B_{\mathrm{nj}}^{\left(R\right)} \end{array}$$ (29)

where the sums run over spin interactions and relaxation mechanisms; $B_{\mathrm{nj}}^{\left(H\right)}$ is a basis set of superoperators spanning the space of relevant Hamiltonians; $B_{\mathrm{nj}}^{\left(R\right)}$ is a basis set of superoperators spanning the space of relevant relaxation superoperators; and $\varphi_{\mathrm{nkj}}^{\left(H,R\right)}$are their phantoms, arrays of coefficients, one for each Voronoi cell of the mesh, indicating how strong the corresponding interaction or relaxation mechanism is in that particular cell. This is a straightforward extension of the notion of phantom from MRI.

**Fig. 5.**
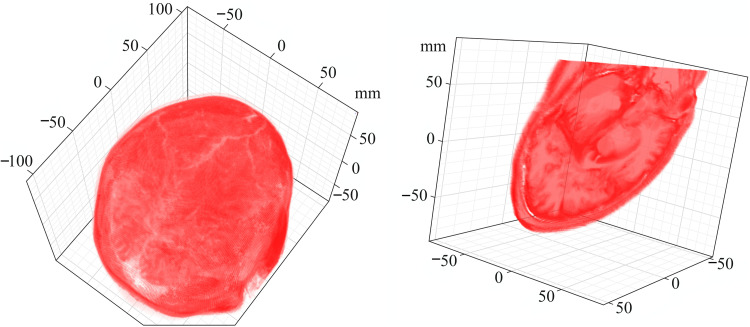
3D relaxation rate phantom used in the MRI example set of Spinach. The volumetric plot in the left panel shows the 3D (216 voxel by 180 voxel by 180 voxel grid) distribution of transverse relaxation rates (mapped into opacity) of water protons in a human brain. Water in blood vessels and cerebrospinal fluid pools appears transparent because it has slower transverse relaxation. The right panel shows a magnetization excitation slice (after a Gaussian radio-frequency pulse and an echo stage under a magnetic field gradient) with the proton density distribution within the slice mapped into opacity. Bone tissue appears transparent because it has lower proton concentration.

The superoperators in Eq. 29 are generated by Spinach on user request; their phantoms are provided by the user. For example, the following Spinach syntax is used (3D echo planar imaging example file, Fig. 5) to specify longitudinal and transverse relaxation phantoms in an MRI simulation

%Phantom library call and sample settings

[R1_Ph,R2_Ph,PD_Ph,dims,npts]= phantoms('brain-highres');

parameters.dims = dims; parameters.npts = npts;

% Relaxation phantom

[R1,R2] = rlx_t1_t2(spin_system);

parameters.rlx_op = {R1,R2};

parameters.rlx_ph = {R1_Ph,R2_Ph};

% Initial and detection state phantoms

parameters.rho0_ph = {PD_Ph};

parameters.rho0_st = {state(spin_system,'Lz','1H')};

parameters.coil_ph = {ones(prod(parameters.npts,1))};

parameters.coil_st = {state(spin_system,'L+','1H')};

In the first paragraph, 3D phantoms (longitudinal relaxation rate, transverse relaxation rate, and proton density) are requested from the phantom library. The second paragraph requests relaxation superoperators Spinach and matches them (rlx_op) to their phantoms (rlx_ph). In the second paragraph, the initial state (rho0_st) is set to longitudinal magnetization weighted by the proton density in three dimensions (rho0_ph). The detection state is set to $L_{+}$ uniformly across the sample.

Simulations involving diffusion and flow additionally have a kinetics phantom: an array of coefficients in front of chemical kinetics generators in every Voronoi cell of the sample. This phantom is updated during the simulation because (as discussed in the “Chemical Transport of Nuclear Spin State” section) chemical transport rates for nuclear spin states are concentration-dependent. Velocity maps and diffusion tensor maps are provided in a similar way: as arrays of vectors or tensors in every voxel or Voronoi cell.

A unique feature of Matlab when applied to physical sciences is that annotated code is often shorter and easier to understand than its verbal description; the code implementing the content of this section is available on GitHub under the MIT license in versions 2.11 and later of Spinach package.

### Solver, concentration stage

The first stage of the simulation involves spatial and chemical degrees of freedom. Their dynamics does not depend on the nuclear spin state and may, therefore, be precomputed by solving the corresponding system of differential equations in the time domain

$$\frac{\partial c_{n}^{\left(k\right)}}{\partial t}=\sum_{m}F_{\mathrm{km}}c_{n}^{\left(m\right)}+f_{n}\left(c_{1}^{\left(k\right)},\ldots ,c_{N}^{\left(k\right)}\right)$$ (30)

where the $c_{n}^{\left(k\right)}$ is the concentration of substance n in Voronoi cell k , F is the transport matrix obtained as described in the “Discretization of the equation of motion” section, and $f_{n}\left(c_{1}^{\left(k\right)},\ldots ,c_{N}^{\left(k\right)}\right)$ is the right-hand side of the mass action law in Eq. 1 describing the kinetics of substance n.

For elementary liquid-phase chemical reactions, the right-hand side of Eq. 30 is always either a linear, or bilinear, or quadratic polynomial function of concentrations. This system is, therefore, well behaved and may be solved using standard methods, for example, Runge-Kutta (*106*, *107*). For aesthetic reasons (chemical kinetics is a Lie semigroup action), we use Lie group solvers (*93*, *94*, *104*). The result is the time dependence $c_{n}^{\left(k\right)}\left(t\right)$ of the concentration of each substance in each Voronoi cell of the mesh. These concentrations determine the rate multipliers in front of the chemical transport generators in Eq. 10 and allow us to proceed to the spin dynamics part.

### Solver, nuclear spin stage

Once the concentration dynamics is known from solving Eq. 30 and assumed to be nuclear spin state independent, Eq. 23 is reduced to a form in which the evolution generator depends on time and location, but not on the concentration-weighted density matrix. Our remaining tasks are to assemble the combined generator matching the state vector structure discussed in the “Generator and state vector structure” section, to run the time evolution, and to project out the observable quantities at each spatial location.

The principal challenge here is astronomical matrix dimensions: For the reactions discussed in the examples below (27 proton spins), even the reduced Liouville space has dimension exceeding 30,000. When this is combined with diffusion and flow across tens of thousands of Voronoi cells (the modest case of the microfluidic chip shown in the right panel of Fig. 1 has 28,902 cells), the combined dimension of the problem goes into billions and becomes intractable even with sparse matrix arithmetic on the strongest existing graphics processing units (GPUs). Thankfully, a workaround was recently published (*71*) that uses relations of the following type (*108*)

$$\left[A⊗B\right]v=\text{vec}\left[{\text{BVA}}^{T}\right]$$ (31)

where A , B , and V are matrices; v is a vector; and vec stands for vectorization, a columnwise reshape of a matrix into a vector. The matrix V is obtained by the reverse procedure: cutting up the column vector v into strips of appropriate size and concatenating them in the horizontal dimension. For large matrices A and B , the right-hand side of Eq. 31 requires significantly less memory because the Kronecker product A⊗B is never computed explicitly. This method may be extended to linear combinations of Kronecker products of any number of matrices (*71*)

$$\begin{array}{c} \left(\alpha \left[A⊗B⊗\ldots \right]+\beta \left[C⊗D⊗\ldots \right]+\ldots \right)v=\\ =\alpha \left[A⊗B⊗\ldots \right]v+\beta \left[C⊗D⊗\ldots \right]v+\ldots \end{array}$$ (32)

and thus to any evolution generator within the remit of this work. Spinach includes a dedicated object that pretends (to Matlab) to be a matrix, but instead buffers linear combinations of unevaluated Kronecker products for the purposes of running Eq. 32 every time its action on a vector is needed. With this technicality out of the way, we proceed in the following stages at each step of a time-domain simulation of the combined dynamics:

1) Use precomputed concentrations of each substance in each Voronoi cell of the mesh to calculate chemical kinetics superoperators in each cell using Eq. 7. Concatenate the superoperators into a block-diagonal sparse matrix matching the state vector structure in Eq. 25.

2) Use interaction and relaxation phantom information to assemble spin Hamiltonian commutation superoperators and relaxation superoperators in each cell of the mesh. Concatenate the superoperators into a block-diagonal sparse matrix matching the state vector structure in Eq. 25. Elementary spin operators are not location- or time-dependent (only their coefficients are) and may, therefore, be precomputed.

3) Assemble the full evolution generator by adding up the kinetics superoperator from item 1, spin evolution generators from item 2, and the transport generator F⊗1 that influences location coordinates but has no effect on the nuclear spin state.

4) Call the time propagation function (step.m in Spinach) that calculates the exponential action of the evolution generator on the state vector without explicitly exponentiating the generator using one of the many variations of the Krylov method [see section 4.9.6 in (*109*)].

5) Use Eq. 28 with precomputed (using user-supplied $b_{+}$ maps) detection state vectors to calculate appropriate observables.

Polyadic objects (*71*, *110*) should be used whenever possible to avoid storing, manipulating, and acting by identical copies of operators. In all but the smallest cases, the use of FP64-capable GPUs is essential because array dimensions go into many millions; Spinach does that on user request. Computational complexity scaling benchmarks on contemporary hardware are presented and discussed in section S1 of the Supplementary Materials.

## EXAMPLES AND BENCHMARKS

In this section, we present practical applications of the formalism built above in the order of increasing feature complexity: from individual processes (diffusion, flow, kinetics, and spin evolution), to their combinations, and then to composite simulations involving all types of dynamics simultaneously.

### Nonlinear kinetics

**Fig. 6.**
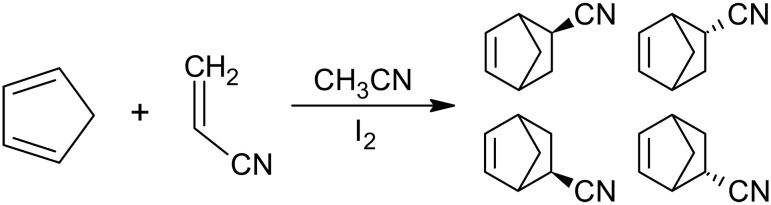
Diels-Alder cycloaddition reaction of 1,3-cyclopentadiene and acrylonitrile. The reaction yields two enantiomeric pairs of bicyclo[2.2.1]hept-5-ene-2-carbonitrile isomers that may be distinct in situations when spin state distribution in the reagents does not follow their molecular symmetry.

A convenient class of second-order reactions that can run in seconds to minutes and, therefore, may be followed in real time by NMR is Diels-Alder cycloaddition (*111*). We consider the reaction between 1,3-cyclopentadiene (A) and acrylonitrile (B) that yields two enantiomeric pairs of bicyclo[2.2.1]hept-5-ene-2-carbonitrile isomers (*112*). We call them endo- and exo-norbornene carbonitrile and denote them (C) and (D), respectively (Fig. 6). Although chemically identical in achiral environments, the enantiomers may still have to be declared as distinct products in the reaction specification (see the “Discretization of the equation of motion” section) because the initial cyclopentadiene may, in principle, have a nonsymmetric nuclear spin state. We use symmetric initial spin states and, therefore, the reaction products are endo- and exo-norbornene carbonitrile

$$\begin{array}{l} \left[A\right]+\left[B\right]\overset{k_{1}}{\to }\;\left[C\right]\\ \left[A\right]+\left[B\right]\overset{k_{2}}{\to }\;\left[D\right] \end{array}$$ (33)

The rate equations may be written in a form that is not commonly used in chemistry textbooks but presents an instance of the Lie equation (*113*) with a state-dependent evolution generator that fits neatly into the algebraic form imposed by Eq. 23

$$\begin{array}{c} \frac{d}{\mathrm{dt}}\left(\begin{array}{c} \left[A\right]\\ \left[B\right]\\ \left[C\right]\\ \left[D\right] \end{array}\right)=\left(\begin{array}{cccc} -\left(k_{1}+k_{2}\right)\left[B\right] & 0 & 0 & 0\\ 0 & -\left(k_{1}+k_{2}\right)\left[A\right] & 0 & 0\\ 0 & k_{1}\left[A\right] & 0 & 0\\ 0 & k_{2}\left[A\right] & 0 & 0 \end{array}\right)\left(\begin{array}{c} \left[A\right]\\ \left[B\right]\\ \left[C\right]\\ \left[D\right] \end{array}\right) \end{array}$$ (34)

**Fig. 7.**
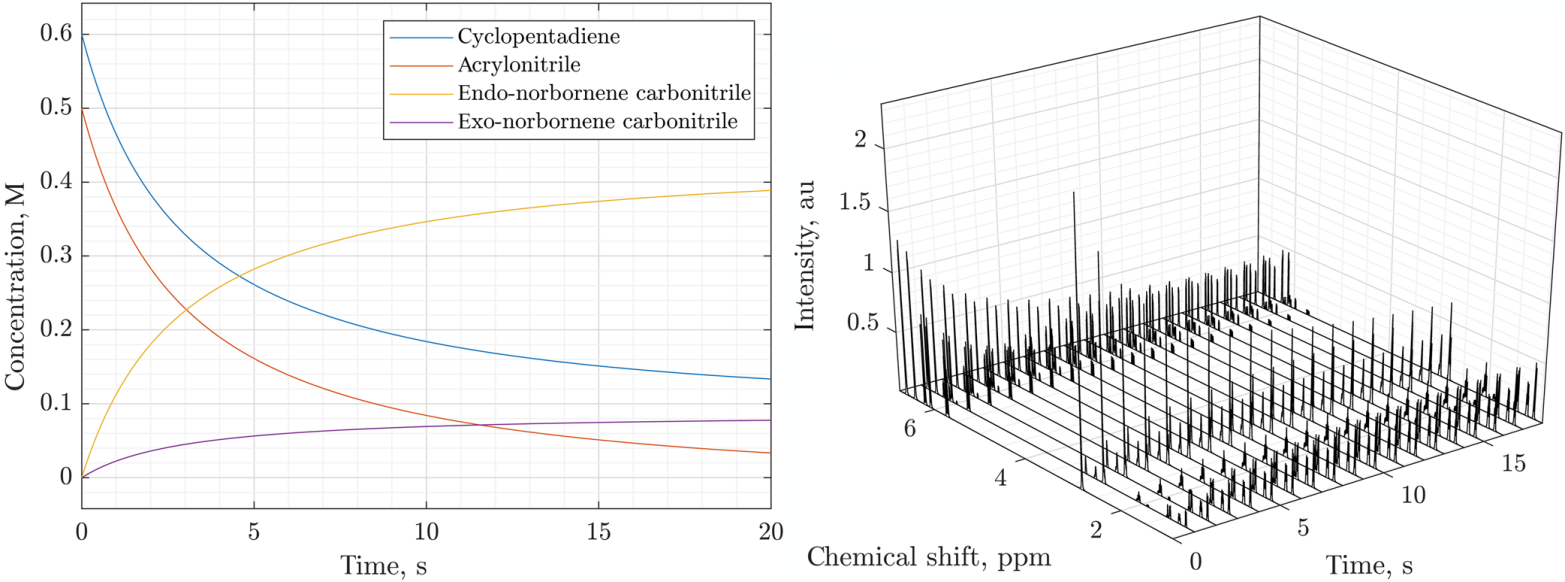
Simulated 600-MHz ^1^H NMR spectra of a second-order reaction system. The left panel shows an example solution of the rate equations describing the reactions in Eq. 33 and Fig. 5 with *k*_1_ = 250 mol/(liter·s), *k*_2_ = 50 mol/(liter·s), *A*_0_ = 0.6 M, *B*_0_ = 0.5 M, *C*_0_ = *D*_0_ = 0. The solution was obtained using a state-dependent geometric integrator (*93*, *104*) because it is compatible with subsequent spin evolution calculations, but any standard method [e.g., Runge-Kutta (*106*, *107*)] may also be used at this stage. The right panel shows the simulated kinetic profile corresponding to a small flip angle pulse-acquire NMR experiment being performed on the reaction mixture every second for 18 s. The simulation was done by time-domain propagation, including Bloch-Redfield-Wangsness relaxation theory, of the concentration-weighted density matrix in Liouville space as described in the main text. Distances (for dipolar interaction tensors) and chemical shielding tensors required by the relaxation superoperator were estimated using density functional theory (GIAO M06/cc-pVTZ in SMD chloroform using Gaussian16), and rotational correlation times were estimated using Stokes-Einstein equation. au, arbitrary units; ppm, parts per million.

An example solution, obtained using the state-dependent geometric integrator introduced in (*96*) and already available in Spinach (*109*), is given in the left panel of Fig. 7. At this point, we have textbook chemical kinetics (*114*) that serves as a unit test on the way to the more complicated composite dynamics cases below.

### Diffusion and flow

**Fig. 8.**
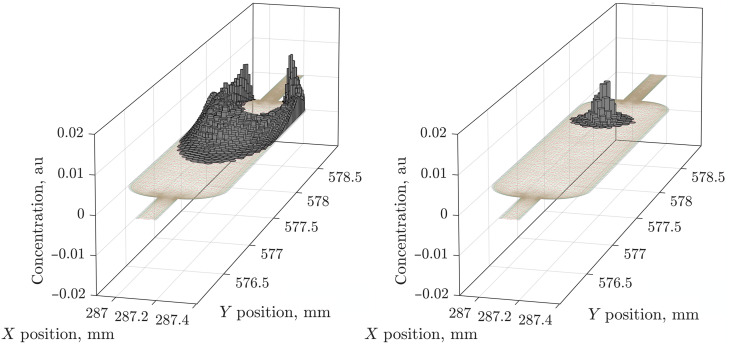
Stop-frames of flow and diffusion simulations. The sample chamber belongs to the microfluidic chip in Fig. 1 (simulation and visualization scripts are included with the example set of Spinach 2.11 and later). Substance concentrations in each Voronoi cell are shown as heights of the gray columns. (**Left**) Flow under the stationary velocity field computed by COMSOL with the initial concentration set to a nonzero value in the distal pipe. (**Right**) Diffusion from a nonzero concentration in a single Voronoi cell in the middle of the chip. Full videos are in the Supplementary Materials. au, arbitrary units.

The other textbook limit is pure spatial transport without kinetics or quantum dynamics, corresponding to Eq. 22 running forward in the time domain through a particular mesh of Voronoi cells. This is standard (*99*, *100*) and, therefore, used as a unit test in this work; Fig. 8 shows two examples: flow through the sample chamber of a microfluidic chip with a no-slip boundary condition (left panel) and diffusion from a localized initial condition on the same grid. The velocity profile was imported from COMSOL; the mesh was preprocessed as described in the “Mesh and flow velocity data handling” section.

### Nonlinear kinetics with spin evolution

We now come to the boundary of the published prior art (*21*), a combination of nonlinear kinetics and spin dynamics that goes beyond what may be described by Bloch equations, the improvement being that our formalism is numerically stable. At this point, the procedures described in the “Chemical transport of nuclear spin state” and “Chemical reaction specification” sections must be performed for the particular cycloaddition reaction discussed in the “Nonlinear kinetics” section.

**Fig. 9.**
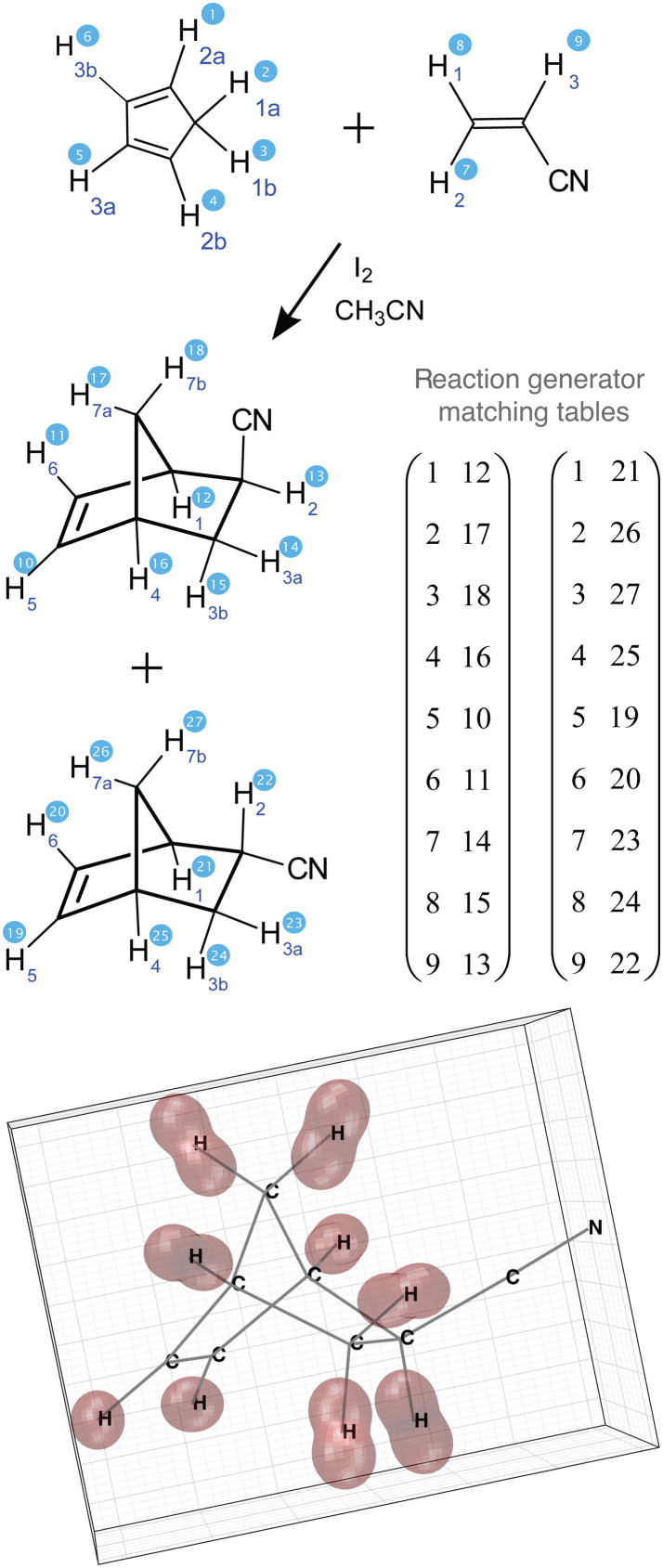
Reaction generator matching table construction example. The Diels-Alder cycloaddition can here proceed in two different ways: one leading to endo-isomer and the other to exo-isomer of norbornene carbonitrile. Accordingly, two matching tables are needed for which spin on the left-hand side of the reaction arrow becomes which spin on the right-hand side. The bottom panel shows a visual representation of the absolute proton chemical shielding tensors in exo-norbornene carbonitrile. Those were obtained as described in the main text and used for the calculation of relaxation superoperators. The visualization (implemented in Spinach 2.11 and later) uses the spherical harmonic representation described in section 3.3.4 of (*15*).

The necessary spin indexing is illustrated in Fig. 9. In the Spinach input script, all reactants and products are specified in the same input stream and then partitioned into sets of spins belonging to individual substances as described in the “Chemical reaction specification” section: spins 1 to 6 for cyclopentadiene, 7 to 9 for acrylonitrile, and so on. The matching table shown in Fig. 9 is then supplied; it tells the reaction generator function which spin on the left side of the reaction arrow becomes which spin on the right in each chemical process. We have two reactions, one producing exo- and the other endo-isomer of norbornene carbonitrile, and, therefore, two matching tables.

The spin evolution generator is composed of the Hamiltonian commutation superoperator (we have used experimentally determined chemical shifts and *J*-couplings, see section S4 of the Supplementary Materials) and the relaxation superoperator that was obtained using Bloch-Redfield-Wangsness theory. Its numerical implementation technicalities are reported elsewhere (*115*, *116*); we have included dipolar relaxation, chemical shift anisotropy relaxation, and their cross-correlations. Molecular geometries and anisotropic parts of the chemical shielding tensors were obtained from density functional theory calculations (GIAO M06/cc-pVTZ in SMD acetonitrile) using Gaussian (*117*–*121*) and imported into Spinach. Proton shielding tensors for one of the products are shown in the bottom panel of Fig. 9.

The use of quantum state transport generators is best illustrated using Matlab code. We first send reaction specifications described in the “Chemical reaction specification” section to the generator build script

% Reaction generators

G1 = react_gen(spin_system,reaction{1});

G2 = react_gen(spin_system,reaction{2});

Here, spin_system is the global object used by *Spinach* to store spin system information (*105*). The resulting generator variable is a cell array of matrices, one per reactant, draining and mapping each basis state in the reactant state space into its destination in the product state space.

The kinetic terms of Eq. 23 may now be built. The state vector is concentration-weighted—for each reactant, its own concentration is already in the state vector—and, therefore, only concentrations of the other species should be present in the coefficients multiplying the generators

% Build the composite evolution generator

F=H+1i*R+1i*k1*G1{1}*B(t) ... % Reaction 1 from substance A

+1i*k1*A(t)*G1{2} ... % Reaction 1 from substance B

+1i*k2*G2{1}*B(t) ... % Reaction 2 from substance A

+1i*k2*A(t)*G2{2}; % Reaction 2 from substance B

In this expression, concentrations come from the preceding calculation (see the “Solver, concentration stage” section) of kinetics and spatial transport. Note the absence of concentration denominators everywhere: This formalism is numerically stable at low concentrations. At this point, the evolution generator assembly is finished and its exponential action may be used to propagate the system forward in time.

The result is shown in the right panel of Fig. 9; this is the simplest calculation that requires the use of the two-stage process described in Implementation details: first, the concentrations, and, then, the spin dynamics using those concentrations as known functions of time. The first stage was discussed above, and the structure of the second stage time loop is best illustrated with Matlab code

% Preallocate the trajectory and get it started

traj=zeros([numel(eta) nsteps+1]); traj(:,1)=eta;

% Run evolution

for n=1:nsteps

% Build the left interval edge composite evolution generator

F_L=1i*k1*G1{1}*B(time_axis(n))...

+1i*k1*A(time_axis(n))*G1{2}...

+1i*k2*G2{1}*B(time_axis(n))...

+1i*k2*A(time_axis(n))*G2{2};

% Build the right interval edge composite evolution generator

F_R=1i*k1*G1{1}*B(time_axis(n + 1))...

+1i*k1*A(time_axis(n + 1))*G1{2}...

+1i*k2*G2{1}*B(time_axis(n + 1))...

+1i*k2*A(time_axis(n + 1))*G2{2};

% Take the time step using the two-point Lie quadrature

traj(:,n+1)=step(spin_system,{F_L,F_R},traj(:,n),dt);

end

Here, time runs on a finite grid on which the spin evolution trajectory is then calculated using the two-point Lie group integrator (*93*, *104*) that was recently implemented into *Spinach* (*96*).

### Diffusion and flow with spin evolution

This special case is well researched and covered in the literature: diffusion MRI for Bloch equation models (*9*, *122*), diffusion-ordered NMR spectroscopy for large coupled spin systems (*123*, *124*), rotational (*125*, *126*) and translational (*127*) diffusion as relaxation mechanisms, material porosity characterization by long-lived state diffusion measurements (*128*), etc. The mathematics here is a straightforward direct product of spin and location degrees of freedom (*70*, *126*), its implementation in Spinach has already been discussed elsewhere (*129*, *130*). The only major numerical simulation problem in these settings, large matrix dimensions, has recently been solved (*71*). For our purposes here, this is a unit test on the way to more complicated simulations.

**Fig. 10.**
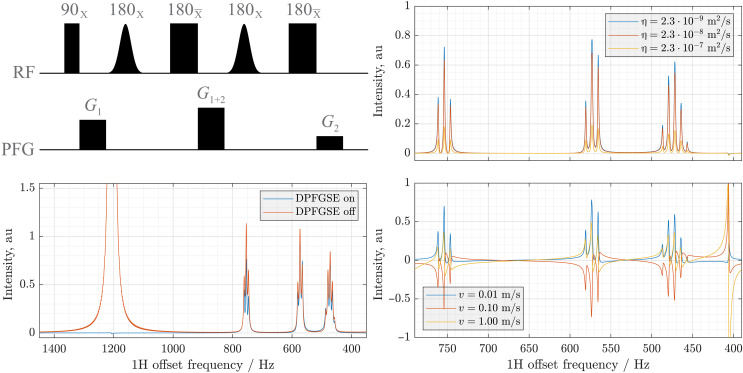
Double pulsed field gradient spin echo pulse sequence and its simulated performance. The system is gamma-aminobutyric acid (GABA, six *J*-coupled spins) dissolved in 95% D_2_O, with the spatial dynamics (diffusion and flow) calculations performed on an explicit spatial grid representing a 15 mm long NMR sample as described in the main text. (**Top left**) Double pulsed field gradient spin echo (DPFGSE) pulse sequence; selective Gaussian pulses are applied to the water signal. (**Bottom left**) 250-MHz ^1^H NMR spectrum of gamma-aminobutyric acid with (blue line) and without (red line) DPFGSE water signal suppression; note the attenuation of the signal at 1200 Hz. (**Top right**) Signal attenuation resulting from incomplete magnetization refocusing, as a function of the diffusion coefficient; blue line corresponds to water at room temperature. (**Bottom right**) Signal phase errors resulting from the presence of hydrodynamic flow with indicated velocities. The artefact at 400 Hz is a reflection of the incompletely suppressed water signal. au, arbitrary units; RF, radiofrequency; PFG, pulsed field gradient.

In the context of dynamics of multispin systems, a good illustration of the implementation reported here is selective suppression and excitation of NMR signals using the double pulsed field gradient spin echo (*131*) pulse sequence (Fig. 10, top left). This is one of the best solvent signal suppression methods because its mechanism is resistant to gradient spiral refocusing errors introduced by diffusion (Fig. 10, top right). Here, spatial dynamics is generated by the first derivative operator with respect to location (hydrodynamic flow) and second derivative operator (translational diffusion); both were represented by finite difference operators generated as described in (*71*) using seven-point stencils on a 500-point grid for a 15-mm-long sample with a periodic boundary condition.

The simulations were done for a 5.87-tesla magnet (250 MHz proton frequency), using 0.10 T/m (*G*_1_) and 0.15 T/m (*G*_2_) pulsed field gradients of 1.0-ms duration, explicit 20-ms Gaussian soft pulses with 1220 Hz offset, nutation frequency of 1700 Hz, and 10 discretization slices. Diffusion coefficients and flow velocities were varied as shown in Fig. 10. Physically correct outcomes are seen: accelerating diffusion (a spatially symmetric process) causes magnetization losses but no artefacts (top right panel), whereas accelerating the flow (a nonsymmetric process) degrades solvent suppression performance and generates phase distortions due to incomplete refocusing of the gradient spirals.

### Diffusion and flow with nonlinear kinetics and spin evolution

This is our final destination; all processes described in the previous sections are now simultaneously active. The principal problem here is that the combined dimension of spin evolution generators for the cycloaddition reaction [30,466 in the IK-2 basis set (*85*)] and spatial dynamics generators for diffusion and flow (2659 cells for the mesh shown in Fig. 9) is close to 100 million. This makes the polyadic representation for the combined evolution generator (*71*) unavoidable. Strong GPUs (we use a server with eight Nvidia H200 cards) are also recommended.

**Fig. 11.**
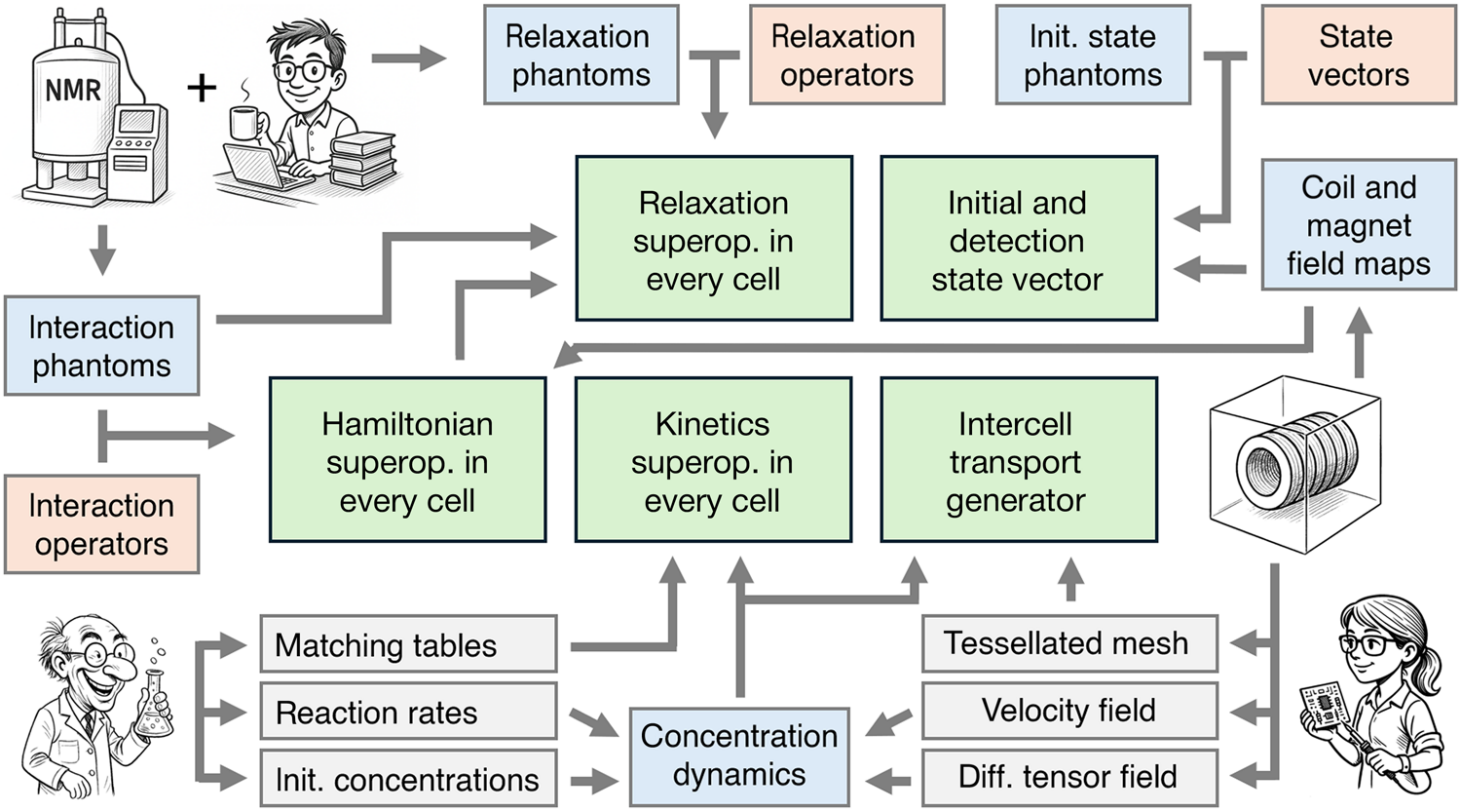
Overview of the code flow in the microfluidics module of Spinach. There are five types of phantoms (blue squares) that specify spatial distributions of simulation components: interaction (e.g., pulsed field gradients), relaxation (e.g., tissue type), initial state (e.g., initial magnetization distribution), coil field map (obtained, along with the flow velocity field, from separate COMSOL simulations), and concentration (e.g., chemical reactions and spatial transport). These phantoms are combined with their corresponding Liouville space superoperators as described in the main text to produce the overall system evolution generator at each time step in the simulation. Concentration dynamics is precomputed separately and then used as a set of time-dependent parameters in the spatially distributed spin dynamics simulation. Any similarity between the cartoon characters and any of the authors is absolutely intentional.

The calculation proceeded in the following stages (a schematic is given in Fig. 11, and the process is automated in Spinach 2.11 and later versions):

1) Concentration dynamics simulation from the user-specified initial concentration distribution (small amounts of cyclopentadiene and acrylonitrile in adjacent regions of the reaction chamber, fig. S4 in the Supplementary Materials) in the presence of kinetics and spatial motion.

Diffusion and flow evolution generator F is built as described in the “Discretization of the equation of motion” section, the polynomials $f_{n}\left(c_{1}^{\left(k\right)},\ldots ,c_{N}^{\left(k\right)}\right)$ responsible for the reaction kinetics are assembled from the reaction descriptors (see the “Chemical reaction specification” section) provided by the user.

The two-point Lie group method (*93*–*95*, *104*) implemented in Spinach step.m function (*96*) is then used to solve Eq. 30 and obtain the time dependence of all concentrations in all Voronoi cells of the mesh (fig. S4 in the Supplementary Materials).

An array of Matlab griddedInterpolant objects (one for each substance in each cell) is then created for concentrations. Their role is to interpolate time: The time grid used by the subsequent spin dynamics simulation stage is not necessarily the same.

2) Spin dynamics infrastructure assembly that must now account for the fact that some terms in Eq. 23 may be time-, location-, and concentration-dependent.

Chemical reaction generators are built as described in the “Chemical transport of nuclear spin state” section. At this point, those are generators in the Lie semigroup sense; they will be multiplied by appropriate coefficients and exponentiated when the time evolution loop is computed.

Superoperators of all pertinent individual interactions (including Cartesian spin operators for use in pulses and pulsed field gradients) are then requested from Spinach kernel. Their coefficients will also be decided when the time evolution loop starts.

All pertinent spin state vectors are requested from Spinach kernel; the initial state is assembled in every cell of the mesh by multiplying these vectors by appropriate concentrations as per Eq. 25. Detection states are built using the same state vectors, but using coil $b_{+}$ maps as coefficients as per Eq. 27—coils see and affect different locations differently (*12*, *72*).

3) Time evolution loop must, at each step, apply the various time-, location-, and concentration-dependent coefficients to the operators built above, assemble the evolution generator, and propagate the system forward in time. At each time step, the following events take place.

Diffusion and flow generator matrix F is inherited from stage 1. Spatial dynamics is assumed to be nuclear spin independent; a Kronecker product with a unit spin superoperator must, therefore, be taken. In practice, F⊗1 is stored as a polyadic object and the Kronecker product is never computed explicitly. If F is time-independent, it may be precomputed.

Hamiltonian, relaxation, and kinetics superoperators are assembled in each cell of the mesh with appropriate time- and location-dependent coefficients describing radio frequency pulses, pulsed field gradients, concentrations, etc. This completes the building of spin evolution generators in each cell of the mesh; time-independent terms may also be precomputed.

Spin evolution generators pertaining to individual cells of the mesh are concatenated into a block-diagonal matrix. Location-independent terms may be stored as 1⊗H and 1⊗Rpolyadic terms with unopened Kronecker products. This completes the construction of the global evolution generator acting on the column of concentration-weighted state vectors in Eq. 23; a time step may now be taken. At this point, typical evolution generator matrix dimensions are in the millions (thousands for the mesh cell count kroneckered with thousands for the spin state space)—what saves us from a memory overflow is the fact that both sets of matrices are very sparse and some are stored in a polyadic format. Exponentiating such an object explicitly is out of the question: The time step must instead be computed using a Krylov-like method, and we recommend the one described in section 4.9.6 of Kuprov’s book (*109*). The propagated state vector may either be stored for later use, or observables may be computed at this point using Eq. 28 if memory efficiency is a concern: Unlike the evolution generators, the trajectory array is not sparse.

**Fig. 12.**
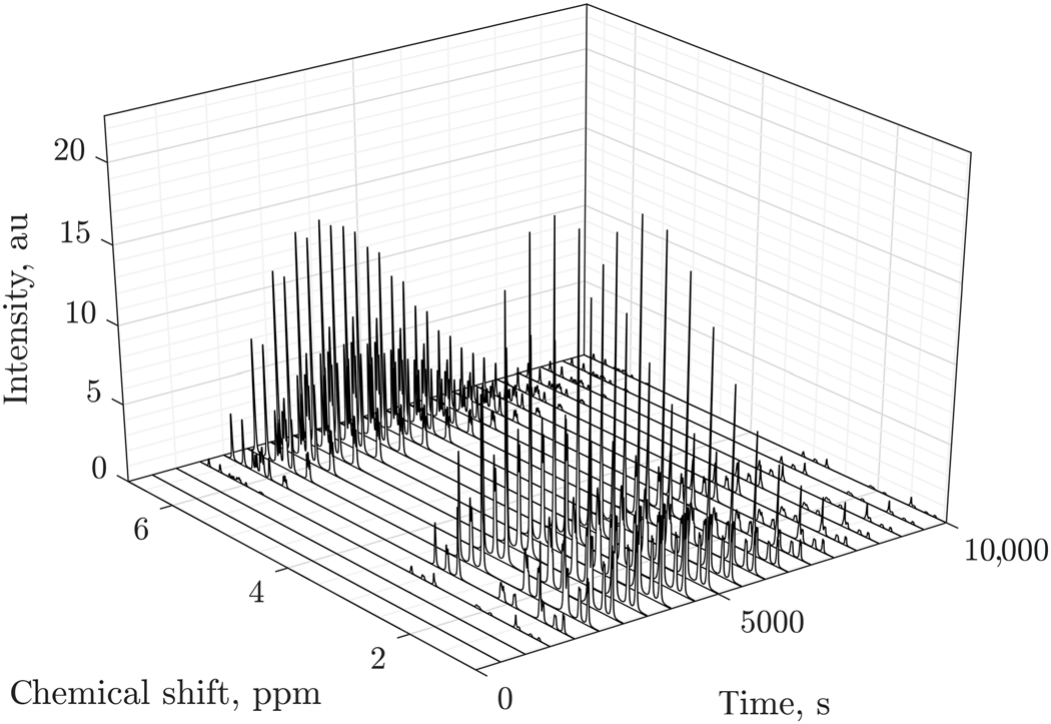
Simulated 600 MHz ^1^H NMR spectra of a flowing and diffusing second-order reaction system. Diels-Alder cycloaddition (acrylonitrile + cyclopentadiene into endo- and exo-norbornene carbonitrile, 30 proton spins in total) as excited and then detected at regular intervals by a radio-frequency coil located between 238.0 and 238.3 mm X position, and between 577.0 and 577.5 mm Y position in the reaction chamber of the microfluidic chip shown in Fig. 8. Reagents are initially located outside the coil, the signals appear as they flow in and change relative intensities as the reaction proceeds. The signals then fade to zero as the products and the remaining reagents flow out of the other end of the coil. au, arbitrary units; ppm, parts per million.

At this point, we have a detailed system trajectory with cell-by-cell chemical, hydro-, and spin dynamics and also the observables seen by the radio-frequency coil—the signals in Fig. 12 first rise as chemicals flow into the region where spins are affected and seen by the coil, changing their relative intensity as the chemical reaction proceeds, and then fade as the reaction products and leftover reagents flow out of the coil area. Throughout the process, the quantum mechanical description of spin dynamics is maintained, as evidenced by the *J*-coupling patterns in all NMR signals.

Although we do not discuss visualization logistics (the matter is well researched), considerable further programming and data handling effort is of course needed to present time- and location-dependent NMR observables in a user-friendly way. Those infrastructure functions are supplied with Spinach.

## CONCLUSIONS AND OUTLOOK

A combination of recent developments in spin dynamics [restricted state spaces (*85*) and polyadic evolution generators (*71*)], numerical linear algebra [Lie group integrators (*93*–*95*, *104*) and spectral methods (*132*)], and computer science [sparse matrix libraries (*133*), graphics processing units (*134*, *135*), and specialized scientific computing languages (*97*)] has finally made it possible to perform NMR simulations for nontrivial spin systems in the simultaneous presence of diffusion, hydrodynamics, and nonlinear kinetics. An open-source implementation in Spinach has required a formalism update [to avoid a numerical stability issue in the prior art (*21*)] and considerable software engineering effort; they are described here. As an example, we have used a cycloaddition reaction in a flowing microfluidic chip, for which the simulation is just about feasible on a strong GPU server at the time of writing; it will likely fit into a laptop in a few years’ time.

## Supplementary Material

20251022-1

## Notes

**Correction (6 November 2025):** In the original version of the article, the Matlab code for the second stage loop on pp. 11 and 12 of the PDF became corrupted during typesetting. The code has been corrected in the PDF and HTML versions of the article.

## Supplementary Materials

### The PDF file includes:

Sections S1 to S6 Figs. S1 to S4 Table S1 Legends for movies S1 and S2
