# Supplementary material for: Chemical hydrodynamics of nuclear spin states
Source: Sci Adv. 2025 Oct 22;11(43):eady9103. doi: 10.1126/sciadv.ady9103 (PMC12542940; doi:10.1126/sciadv.ady9103)
Supplement: Supplementary file 2 — Sections S1 to S6 Figs. S1 to S4 Table S1 Legends for movies S1 and S2 [file sciadv.ady9103_sm.pdf]

Science Advances
AAAS

Supplementary Materials for

Chemical hydrodynamics of nuclear spin states

Anupama Acharya *et al.*

Corresponding author: Ilya Kuprov, ilya.kuprov@weizmann.ac.il

Sci. Adv. **11**, eady9103 (2025)
DOI: 10.1126/sciadv.ady9103

The PDF file includes:

Sections S1 to S6
Figs. S1 to S4
Table S1
Legends for movies S1 and S2

Other Supplementary Material for this manuscript includes the following:

Movies S1 and S2

S1. Computational complexity scaling

Simulations described in the main text contain five logistically distinct stages:

(a) Diffusion and flow generator construction described in Section 3.2 of the main text. At this stage, the mesh is analysed and its associated fields (velocity, diffusion, *etc.*) are converted into matrix representations of the corresponding evolution generators. The complexity of this stage is linear with respect to the mesh cell count (Figure S1, blue bars), because the algorithm fills in sparse matrices cell by cell. At the evolution generator level, cells only see their nearest neighbours, hence the overall linear complexity with the number of cells. This stage precedes the spin dynamics simulation and remains the same for any spin system. Its timing is therefore unaffected by the number of spins (Figure S2, blue bars).

(b) Pre-calculation of nuclear spin independent processes: diffusion, hydrodynamics, chemical kinetics. At this stage, Eq (3) of the main text is solved, the complexity is expected to be asymptotically linear with respect to the mesh cell count, but is in practice nearly constant (Figure S1, red bars) because the evolution generators are very sparse and housekeeping tasks (such as GPU communications) dominate the wall clock time. This stage is also the same for any spin system, its timing is therefore unaffected by the number of spins (Figure S2, red bars).

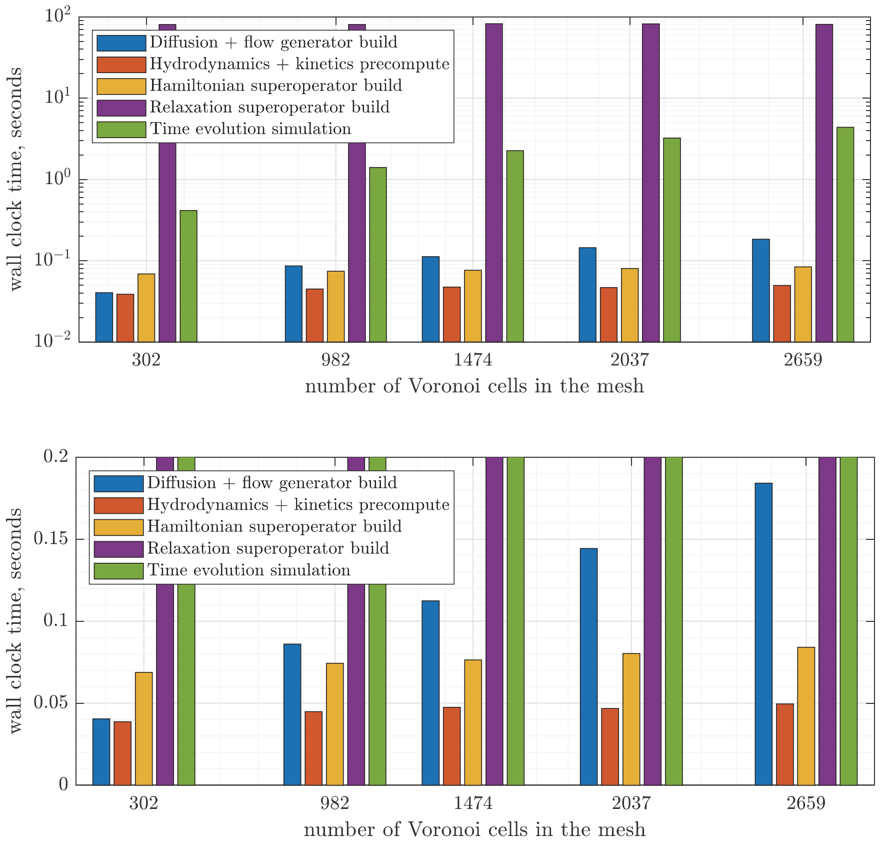

Figure S1. *Wall clock time scaling, with respect to the spatial mesh size, of the five logistically distinct stages of a Liouville-space spin dynamics simulation in the simultaneous presence of diffusion, flow, second-order kinetics, and relaxation (Bloch-Redfield-Wangsness theory). The spin system and the parameters of its spatial and chemical dynamics are described in Section 4 of the main text.*

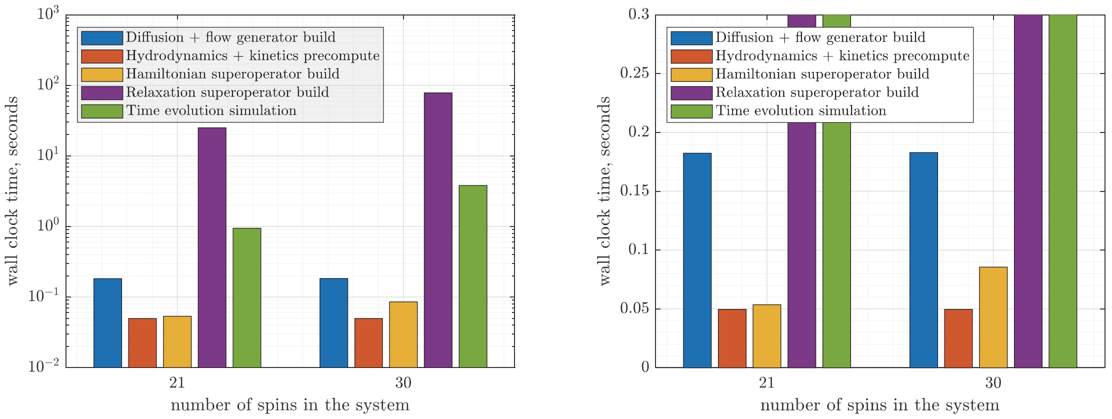

Figure S2. *Wall clock time scaling, with respect to the number of spins, of the five logistically distinct stages of a Liouville-space spin dynamics simulation in the simultaneous presence of diffusion, flow, second-order kinetics, and relaxation (Bloch-Redfield-Wangsness theory). The spin system and the parameters of its spatial and chemical dynamics are described in Section 4 of the main text.*

(c) Hamiltonian superoperator construction and its replication, with appropriate alterations (for example, due to a non-uniform B_0 field), to every cell of the mesh. The complexity of this step scales approximately linearly with the mesh size (Figure S1, yellow bars) because a different instance of the Hamiltonian is associated with each cell of the mesh. It also scales quite steeply (but polynomially when the restricted state space approximation is used), with the number of spins because the dimension of the Hamiltonian superoperator matrix is increased (Figure S2, yellow bars). This stage carries significant housekeeping costs associated with setting up *Spinach* data structures and building the reduced Liouville state space.

(d) Relaxation superoperator construction and its replication, with appropriate alterations (for example, due to variations in local viscosity), to every cell of the mesh. This is a very expensive object (Figure S1, purple bars); the wall clock time is dominated by the numerical evaluation of Redfield's integral, relative to which the subsequent replication cost is negligible. Thus, the principal factor in the complexity scaling is the size of the spin system (Figure S2, purple bars). The scaling was previously been demonstrated to be polynomial when a restricted state space approximation is used; the subsequent mesh cell replication cost is scales linearly with respect to the number of cells in the mesh.

(e) Full spin dynamics simulation using Eq (22) of the main text. The complexity of this stage scales approximately linearly with respect to the number of mesh cells (Figure S1) and polynomially (when the restricted state space approximation is used, Figure S2) with respect to the number of spins in the system.

As expected, the overall complexity scaling is between linear and quadratic with respect to the number of spatial mesh cells. This is because spatial evolution generators are $N_{\text{cells}} \times N_{\text{cells}}$ matrices acting on vectors with an exponential map. For full matrices, the complexity of each time step would be $O\left(N_{\text{cells}}^2\right)$ when the Krylov method is used, but the matrices in question are very sparse. The overall complexity with respect the number of spins is polynomial because the basis set dimension is polynomial and subsequent matrix-vector operations have between linear and quadratic complexity.

S2. Limitations and approximations

This section contains a summary of approximations and assumptions made by the model presented in the main text. This is necessary because its applicability range, although larger than the previous state of the art, is still limited relative to the breadth of the related chemistry and physics.

1. **Nuclear spin independent kinetics and spatial motion** – nuclear spin interactions are assumed to be too weak to influence reactions, diffusion, or flow. Spatial motion affects nuclear spins, but there is no back action. Reaction rate expressions contain only concentrations; they are assumed to be independent of the spin state. The flow is modelled using a user-specified velocity field that is not influenced by spin dynamics.

2. **Closed-shell ground-state electronic structure** – all molecules are assumed to be diamagnetic and to remain in their ground electronic state. Molecular structure rearrangements influence nuclear spins only through effective spin Hamiltonian parameters. The formalism is inapplicable when radical pairs or other open-shell intermediates are present. Kinetic and magnetic isotope effects on reaction rate constants are ignored.

3. **Law of mass action with perfect cell mixing** – reactions are assumed to be elementary and to obey deterministic rate equations, implying perfectly mixed Voronoi cell volumes.

4. **Ideal solution thermodynamics and Fickian diffusion** – activity coefficients are absorbed into effective, position-dependent rate and diffusion constants; chemical potentials are replaced by $RT \ln C$, implying uniform temperature and pressure. Cross-diffusion terms are ignored; diffusion and flow are modelled by an ensemble-averaged PDE that neglects stochastic fluctuations at the single molecule level.

5. **Stationary incompressible flow** – the finite-volume discretisation assumes exact local mass conservation and negligible density variations across the flow field.

6. **Mapping of spin spaces in spatial and chemical transport** – when reactants associate, their direct-sum state spaces are mapped into a direct-product state space; upon dissociation correlations are assumed to be irreversibly lost. Apart from that, nuclei are assumed to retain their spin states and correlations during chemical reactions, diffusion, and flow.

7. **Nuclear spin interaction parameters are static** – chemical shifts, *J*-couplings, and other parameters of the nuclear spin Hamiltonian are assumed not to vary with concentration, temperature, or local microenvironment within each Voronoi cell.

8. **Spin Hamiltonian and relaxation superoperator locality** – spin-spin couplings are only considered between nuclei of the same molecule; long-range dipolar order is ignored. Diffusion coefficients and relaxation theory parameters are obtained from user-supplied phantoms, not through self-consistent heat transport.

9. **Linear response detection with fixed coil maps** – the formalism neglects radiation damping, coil loading changes, and sample heating by radiofrequency irradiation. Coil maps are immutable during the simulation; macroscopic sample magnetisation is assumed to be too small to generate longitudinal or transverse feedback fields.

10. **Low Reynolds number regime with ideal walls** – chemical adsorption, surface relaxation, and susceptibility gradients at channel walls are ignored; boundaries are assumed to influence the system only through velocity and no-flux boundary conditions. Laminar flow is assumed to ensure the validity of the stationary velocity map.

These assumptions specify the regimes in which the formalism described in the main text and its *Spinach* implementation are expected to deliver accurate simulations.

S3. Spinach mesh object diagram

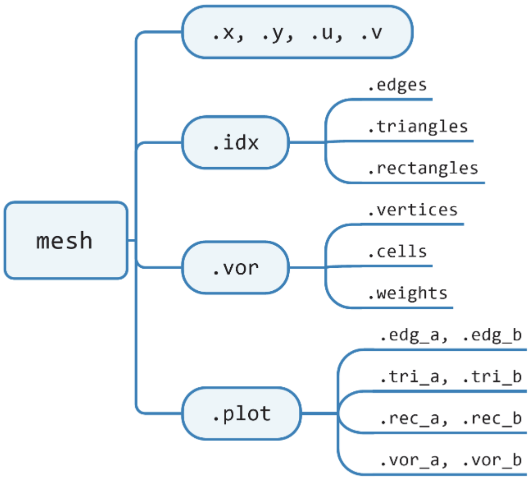

Figure S3. *Spinach 2D mesh data structure schematic, including coordinate and velocity arrays, mesh and tessellation indices, and pre-computed information to facilitate the plotting as discussed in the main text.*

S4. ^{1}H and ^{13}C NMR data for 5-norbornene-2-carbonitrile.

Table S1. *^{1}H and ^{13}C NMR data for 5-norbornene-2-carbonitrile.*

atom	*endo* isomer, 400 MHz, $CDCl_3$		*exo* isomer, 400 MHz, $CDCl_3$	
	^{1}H, ppm	^{13}C, ppm	^{1}H, ppm	^{13}C, ppm
1	3.167	45.7	3.158	47.4
2	2.781	27.1	1.897	27.2
3	2.074	32.4	2.113	32.2
3’	1.263		1.496	
4	2.959	42.3	2.983	41.8
5	6.262	138.8	6.099	138.1
6	6.129	132.6	5.971	134.0
7	1.449	48.4	1.496	47.1
7’	1.133		1.496	
8		123.0		123.6

The full list of *J*-couplings is given in `dac_reaction.m` function of *Spinach*.

S5. Stop-frames from simulation videos

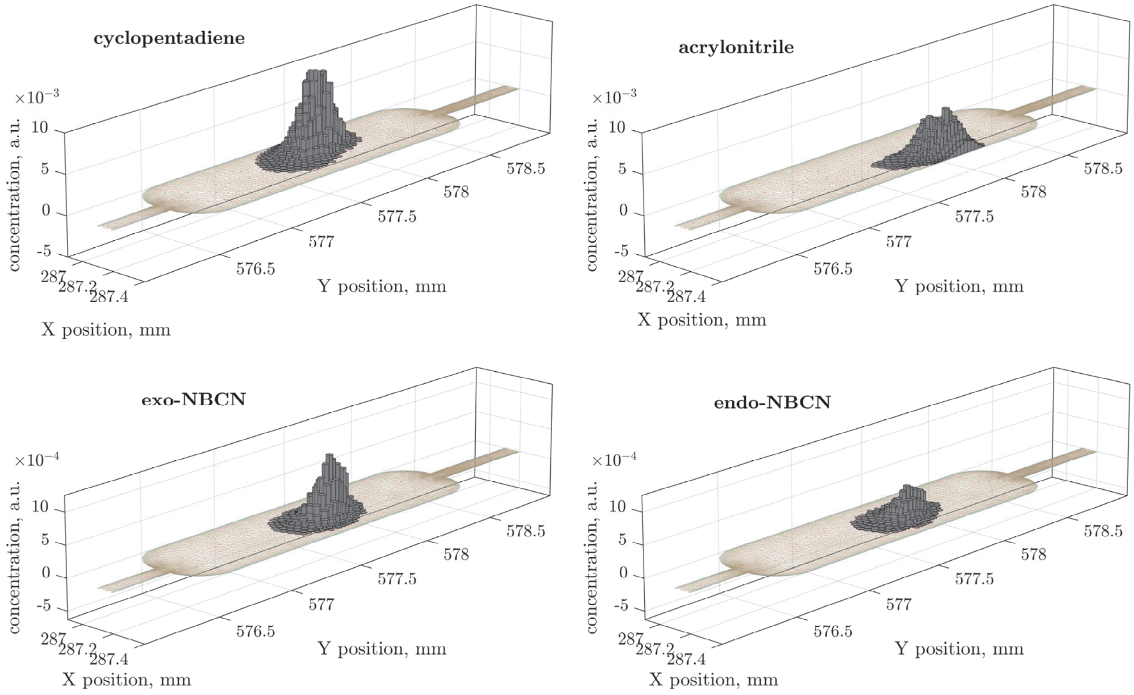

Figure S4. A frame from the spatial dynamics and chemical kinetics simulation stage. The initial condition is drops of cyclopentadiene and acrylonitrile in adjacent regions in the upper part of the chip. As the reagents flow downwards and mix, the reaction produces unequal quantities of exo- and endo-norbornene carbonitrile. The resulting time dependence of all concentrations in all Voronoi cells of the mesh is used in the subsequent stages to generate the non-linear kinetics superoperator at every time point in the spin dynamics simulation.

S6. Captions for simulation videos

Movie S1.mp4

Full video of the simulation presented in the left panel of Figure 6 in the main text: flow under the stationary velocity field computed by COMSOL with the initial concentration set to a non-zero value in the distal pipe.

Movie S2.mp4

Full video of the simulation presented in the right panel of Figure 6 in the main text: diffusion from non-zero concentrations in two arbitrarily chosen Voronoi cells in the middle of the chip.
